# Immobilization of Papain on Chitin and Chitosan and Recycling of Soluble Enzyme for Deflocculation of *Saccharomyces cerevisiae* from Bioethanol Distilleries

**DOI:** 10.1155/2015/573721

**Published:** 2015-01-01

**Authors:** Douglas Fernandes Silva, Henrique Rosa, Ana Flavia Azevedo Carvalho, Pedro Oliva-Neto

**Affiliations:** ^1^Department of Biological Science, University of State of São Paulo (UNESP), 19806-900 Assis, SP, Brazil; ^2^Food Engineering Faculty, State University of Campinas (UNICAMP), 13083-970 Campinas, SP, Brazil

## Abstract

Yeast flocculation (*Saccharomyces cerevisiae*) is one of the most important problems in fuel ethanol production. Yeast flocculation causes operational difficulties and increase in the ethanol cost. Proteolytic enzymes can solve this problem since it does not depend on these changes. The recycling of soluble papain and the immobilization of this enzyme on chitin or chitosan were studied. Some cross-linking agents were evaluated in the action of proteolytic activity of papain. The glutaraldehyde (0.1–10% w·v^−1^), polyethyleneimine (0.5% v·v^−1^), and tripolyphosphate (1–10% w·v^−1^) inactivated the enzyme in this range, respectively. Glutaraldehyde inhibited all treatments of papain immobilization. The chitosan cross-linked with TPP in 5 h of reaction showed the yield of active immobilized enzyme of 15.7% and 6.07% in chitosan treated with 0.1% PEI. Although these immobilizations have been possible, these levels have not been enough to cause deflocculation of yeast cells. Free enzyme was efficient for yeast deflocculation in dosages of 3 to 4 g·L^−1^. Recycling of soluble papain by centrifugation was effective for 14 cycles with yeast suspension in time perfectly compatible to industrial conditions. The reuse of proteases applied after yeast suspension by additional yeast centrifugation could be an alternative to cost reduction of these enzymes.

## 1. Introduction

During the industrial process of fuel ethanol fermentation the contamination caused by bacteria and/or wild yeast is very common. The microbial contaminants cause cells flocculation or flakes of yeast and bacteria and this contamination causes settling of yeast cells at the bottom of the vats. The yeast flocculation is a serious current problem in fuel ethanol technology since this process uses cells recycle. The flocculation of yeast cells decreases the ethanol efficiency by some operational problems as the loss of yeast cells due to difficulties in yeast centrifugation and obstruction of bombs and pipes. Other important problem caused by yeast cells flocculation is the adhesion of bacterial cells on yeast cell surface in the yeast flake. This fact is responsible for the increase of lactic acid bacteria population. Consequently, organic acids are produced leading to yeast metabolism inhibition and ethanol production [1–3]. Furthermore, yeast flocculation increases the use of sulfuric acid and also increases the cost of fuel ethanol [1, 2]. Besides bacteria, wild yeasts and salts could be responsible for the phenomenon of yeast flocculation causing serious operational problems and economic losses in the processes [2–4]. Protein factors associated with minerals such as Ca^+2^ [3, 5], as well as mannans, have been proved to be involved in the process of flocculation.

The cell's flocculation also is responsible for the increase in the production of organic acids by bacteria causing inhibition of yeast metabolism and consumption of sugar by contaminants [6, 7]. All these problems result in a partial conversion of sugar into ethanol and CO_2_ decreasing the ethanol yield and productivity and increasing the use of chemicals like sulfuric acid, antibiotics, and antifoam to control, respectively, yeast flocculation, microbial infection, and bubbles [8–10].

Conventional treatment of flocculated yeast cells using sulfuric acid leads to an immediate cell deflocculation, although this procedure allows the return of the flocculation due to pH change of the industrial process, when yeasts are returned to the fermenter (pH 4.0). The low pH used for yeast deflocculation can affect the yeast metabolism decreasing yeast cell viability, depending on the operational process. The residence times of the acid treatment are generally 0.5 to 2 hours, but an increase of these times and/or decrease of pH cause a detrimental impact on the yeast metabolism. Furthermore, younger and older yeast cells are less resistant to this treatment [11].

The action of proteases has been effective on yeast deflocculation [1]. A widely used proteolytic enzyme is papain, an alkaloid protein from the latex of papaya (*Carica papaya*), which is characterized as a cysteine endopeptidase that has strong proteolytic action [12] and it is relatively inexpensive.

The treatment with proteolytic enzymes for the control of flocculation could be an adequate alternative, since this method is less affected by the changes in the pH of the process [1] and it does not affect the yeast metabolism [13]. However, the application of these enzymes on industrial scale will only be economically viable if they are of low cost. For this reason, the reuse of proteases is required, which can be achieved with the recovery of free enzyme by centrifugation or ultrafiltration in the bioprocess [5, 14, 15] or, alternatively the immobilization of this enzyme on solid supports.

Some low cost products can be used in enzyme immobilization and reuse of papain. The chitin is a natural polysaccharide with acetamide group which has a positive charge [16], and it is possible to be obtained as residue from the food industry [17]. The chitosan is a natural polymer, of low cost, is renewable, and is obtained from chitin by deacetylation with alkali [16, 18]. The lower level of N-acetyl groups (<40%) provides greater solubility when chitosan solutions are in pH below 6.5; this polymer is nontoxic, available in different forms (powder, gel, fibers, and membranes), and easily derivatizable, demonstrating high protein affinity [19, 20]. Therefore, a chemical treatment of chitosan is required in low pH conditions to maintain its insolubility, a key feature of the support for the success of the enzyme immobilization [21]. The treatment of chitosan with sodium tripolyphosphate (Na_5_P_3_O_10_) is necessary to promote the formation of bonds between these molecules reducing its solubility in acid conditions by ionic cross-linking between them [22], preventing chitosan to be dissolved in this condition [23].

The cross-linking method by bifunctional agents (e.g., glutaraldehyde) [19] is one of the most common procedure for immobilization of enzymes since this method is of low cost, simple, and fast and is able to be widely applied [24]. The procedure involves a covalent bond by fixing the enzyme on the matrix or by cross-linking in the matrix, containing the enzyme and a bifunctional agent [4, 25, 26].

In this paper, the reuse of papain through the recovery of soluble enzyme by centrifugation or by its immobilization on polysaccharides was evaluated for* S. cerevisiae* cells deflocculation from fuel ethanol distilleries.

## 2. Materials and Methods

### 2.1. Microorganisms and Reagents

Samples of flocculated* Saccharomyces cerevisiae* from fuel ethanol distillery (Raizen, Maracaí, SP, Brazil) were used in deflocculation test with commercial crude papain (Vetec Química Fina LTDA, EC 3.4.22.2) with 6000 U·mg^−1^ of proteolytic activity. This enzyme was dissolved in phosphate buffer 0.2 mol·L^−1^, pH 6.4. The PA (pure for analysis) reagents used for enzyme immobilization were 25% glutaraldehyde in water, polyethyleneimine (PEI) of high molecular weight (Sigma-Aldrich Co.), and sodium tripolyphosphate (TPP) (Na_5_P_3_O_10_). Chitin was extracted from shrimp shells and high molecular weight chitosan was obtained from Aldrich (code 419419-50 G). The proteolytic activity of papain was determined by hydrolysis of sulfanilamide azocasein [27], and total protein was determined by Bradford [28].

### 2.2. Minimum Inhibitory Concentration of Papain for Yeast Deflocculation

The cells deflocculation of the industrial* S. cerevisiae* was proceeded in a range of 0–4 g·L^−1^ of soluble papain in a 30% (w·v^−1^) flocculated yeast suspension for 15 minutes of reaction, at 27°C, according to a method developed by Ludwig et al. [1] relating absorbance of yeast suspension and percentage of yeast deflocculation (Table 3).

### 2.3. Immobilization of Papain on Chitin or Chitosan

Immobilization of papain on chitin was conducted in two ways [20]: without any pretreatment of the support or pretreatment with 10 mL of 2% (v·v^−1^) glutaraldehyde per gram of chitin at 27°C for 5 h. The enzyme immobilization was conducted by the addition of 1.0 g of treated or untreated chitin into 10 mL of 1% (w·v^−1^) papain solution in phosphate buffer (0.2 mol·L^−1^) and/or by the addition of 0.1% or 0.5% (v·v^−1^) of PEI in papain solution. These protocols were performed in pH 7.0 at 27°C for 12 h in 125 mL Erlenmeyer flasks at shaker 80 rpm, totaling six different immobilization protocols (Table 1).

The cross-linking process of chitosan by sodium tripolyphosphate (TPP) was processed according to Laus et al. [23], in which 1.0 g of chitosan was dissolved into 100 mL of 1% (w·v^−1^) acetic acid solution. This viscous solution was dripped into 1% papain (w·v^−1^) in a ratio of 1 : 2 (v·v^−1^) in three different protocols for 5 or 12 h of reaction (Table 2) in order to evaluate the effect of two different times on the result of immobilization. Subsequently, the immobilized chitin and chitosan were filtered and washed two times with distilled water (100 mL and 1000 mL) and packed in 5°C in phosphate buffer (pH 6.4) for use in testing of the proteolytic activity.

### 2.4. Yeast Deflocculation Using Immobilized Papain

The deflocculation of yeast cells through immobilized papain on chitin or chitosan was conducted in conical glasses with 50 mL of 30% (w·v^−1^) flocculated* S. cerevisiae *from ethanol distillery and 2 g of papain immobilized on chitin or chitosan. The reaction was processed at 25°C and pH 4.5 for 120 min.

### 2.5. Yield Calculations of the Immobilization Process

The yields of enzyme activity and immobilized protein were calculated according to Varavinit et al. [29] by equations

(1) Yield  of  active  immobilized  enzyme  % =Total  activity  of  immobilized  enzyme  A−B×100,

where *A* is total activity of the soluble papain added on the support; *B* is total activity of the enzyme remained in solution after immobilization process. Thus,

(2) $$\begin{array}{c} \text{Yield}\;\text{of}\;\text{immobilized}\;\text{protein}\;\left(\%\right)=\frac{C}{D}\times 100, \end{array}$$

where *C* is total immobilized protein (g); *D* is total protein added on support (g), total protein remaining in solution after the immobilization process (g).

### 2.6. Yeast Viability

The viability of yeast cells was performed by counting living cells by light microscopy in a Neubauer chamber [30], comparing yeast deflocculation by papain (4 g·L^−1^) against H_2_SO_4_ at minimum concentration for complete deflocculation. The mixture was resting for 2 hours. Yeast cells of both test solutions were stained with erythrosine [6]. The yeast viability was expressed by the percentage of live cells of the total number of cells.

### 2.7. Proteolytic Activity and Protein Determination

The proteolytic activity of the enzyme was determined by hydrolysis of azocasein sulfanilamide, according to the method of Leighton et al. [27], at 60°C for 30 minutes. The reaction was stopped with 10% (w·v^−1^) trichloroacetic acid (TCA), and the proteolytic activity was analyzed by spectrophotometer at 440 nm. One unit was defined as the absorbance change in 30 minutes of reaction per mL of solution or g support. The protein was determined according to Bradford [28], and the measurements were performed before and after immobilization procedures.

### 2.8. Recycled Soluble Enzyme in Yeast Deflocculation

The recycle of soluble enzyme was performed according to the best concentration of yeast deflocculation test using soluble papain with or without 0.1 g·L^−1^ sodium dodecyl sulfate (SDS) in 50 mL flocculated yeast cell solution (30% w·v^−1^) in 250 mL Erlenmeyer flask. They were incubated in shaker for 6–120 min at 25°C. After each cycle, 5% of enzyme was replaced by new enzyme in order to maintain the enzyme activity. The quantification of yeast deflocculation and phase separation were evaluated in conical glasses with flocculated* S. cerevisiae *suspension according to Ludwig et al. [1] (Table 3).

### 2.9. Statistic Treatment

The statistical analyses were processed in triplicate of results submitted to analysis of variance (ANOVA), while the means were compared using Student's *t*-test or Tukey test by the program GraphPad InStat version 3.05 (Rutgers University). The treatments were considered statistically significant at *P* < 0.05.

## 3. Results and Discussion

### 3.1. Cell Deflocculation of* S. cerevisiae* by Soluble Papain and Yeast Viability

**Figure 1:**
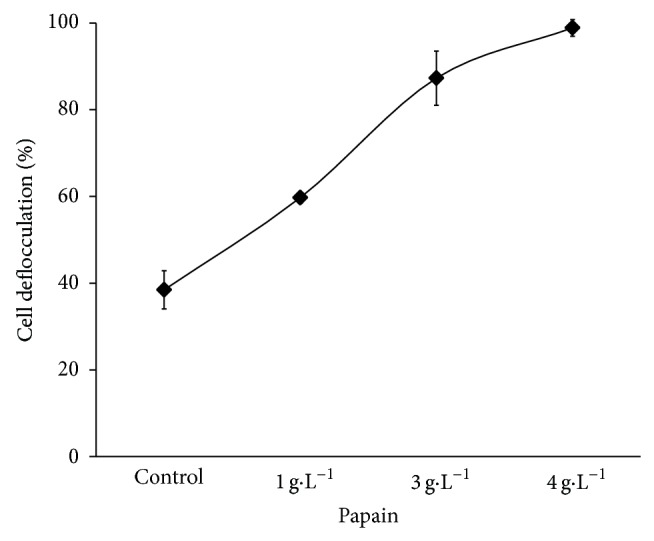
Yeast cell deflocculation on the* S. cerevisiae* suspension from fuel ethanol distillery treated with soluble papain in 15 minutes of reaction.

The minimum concentration of papain necessary to induce yeast cell deflocculation was 3 g·L^−1^ of papain in* S. cerevisiae* suspension after 15 min of reaction (Figure 1), but 4 g·L^−1^ of papain produced better results (ANOVA and Tukey *F* = 162.32, *P* < 0.05) reaching almost 100% of cells deflocculation.

The current method of cell deflocculation of* S. cerevisiae* in industrial fuel ethanol production in Brazil is the use of H_2_SO_4_ in pH 2.5, and this method can decrease yeast cells viability.  Table 4 shows a comparative effect of sulfuric acid and papain (4 g·L^−1^) for 2 h of reaction to induce yeast cell deflocculation. The yeast cells treated with H_2_SO_4_ showed a lesser (*P* < 0.05) yeast viability (67.2%) than papain (72.4%) in just one treatment of 2 h. However, the yeast cells are treated with H_2_SO_4_ about 2-3 times per day, and sometimes pH < 2.5. According to these results papain does not affect cell viability and for this reason it is more convenient than the sulfuric acid method for the health of yeast.

### 3.2. Inhibition of Soluble Papain by Glutaraldehyde, Polyethyleneimine, and Tripolyphosphate

Glutaraldehyde (0.1–10%) strongly inhibited (*P* < 0.05) the papain activity compared to the untreated soluble enzyme. 1–10% sodium tripolyphosphate (TPP) reduced (*P* < 0.05) the proteolytic activity of papain; however, TPP was less aggressive than glutaraldehyde, since 1% TPP reduced only 45% of the enzyme activity, while glutaraldehyde at this same concentration reduced this activity by 94.1%. Despite their inhibition, 1% TPP and 2% glutaraldehyde were evaluated as cross-linking agents in immobilization of papain on chitin (Table 5).

Polyethyleneimine (PEI) also was less inhibitor of papain activity than glutaraldehyde. Up to 84% of proteolytic activity of papain was maintained in concentrations less than 0.5% PEI. However, more than 70% reduction in the papain activity with 1% PEI was verified. Therefore, 0.5% PEI was considered the limit of concentration to use in the tests of immobilization process of papain.

TPP and glutaraldehyde are considered excellent cross-linking agents for chitosan microspheres [31]; however, the inhibitory effect of this agent was proved inducing a total or partial loss of enzyme activity depending on each enzyme and chemical or physical treatments [20]. The action of glutaraldehyde in denaturation of aldehyde dehydrogenase has been showed [32]. The nature of the enzyme is primarily responsible for the denaturing action by glutaraldehyde. Enzymes rich in lysine are more resistant to degrading action of this compound [25]. Therefore, the concentrations of the enzyme and glutaraldehyde need to be carefully considered in order to obtain active derivatives via cross-linking. Low concentrations of enzyme and the bifunctional agent tend to induce intramolecular cross-linking [33]; however, the enzyme activity is inversely proportional to the concentration of glutaraldehyde [34]. Excess of cross-linking can result in a distortion in the enzyme structure [34], and this conformational change may induce loss of the catalytic site, thereby reducing enzyme activity.

### 3.3. Immobilization of Papain on Chitin and Chitosan

#### 3.3.1. Immobilization on Chitin

The process of immobilization of papain on chitin with 0.1% PEI (protocol B) showed better yield of active immobilized enzyme (6%) when compared with protocol A (2.6 times lower), proving the efficiency of PEI in the process of immobilization of papain on chitin. However, although protocol B has shown better performance in yield of active immobilized enzyme, only less than 0.72 U was immobilized on chitin, or 0.11 U/g of support, and only 0.3% of the immobilized protein yield was obtained. Protocol A showed double (0.23 U/g) of support of protein immobilization (Tables 6 and 7).

Depending on the enzyme, the method of chitin treated with glutaraldehyde had a higher immobilization of proteins, probably due to a better cross-link between enzyme-enzyme and enzyme-support [4]. The immobilization of papain on chitin using glutaraldehyde (Tables 6 and 7) has confirmed the inhibition of papain activity in tests with soluble enzyme (Table 5), even if a higher linkage of protein (67.41% or 33.46 mg·g^−1^ support) (Table 7) was obtained. However, only 0.012 U/g of support of the active enzyme was determined. For the protocols with glutaraldehyde (D, E, and F), although they have shown higher yield of immobilized protein than protocols untreated (A, B, and C), all of them did not show yield of active immobilized enzyme (Tables 6 and 7).

This fact could be explained due to the reactivity of glutaraldehyde with some groups of catalytic site of the enzyme, leading to a loss of proteolytic activity [35]. The enzyme was connected to support in various ways and its activity may be affected due to this link involving the catalytic site or preventing its availability to the substrate, leading to a loss of proteolytic activity.

On the other hand, the importance of glutaraldehyde for immobilization process of several enzymes is unquestionable. This agent promotes the formation of Schiff bases between aldehyde and amine groups of support and enzyme, resulting in a better adsorption of the enzyme on the support [4, 20, 36]. Gaspari et al. [36] working with inulinase of* Kluyveromyces marxianus *showed a low yield of immobilization on chitin without any cross-linking agent, and best results occurred when it was previously treated with glutaraldehyde. On the other hand, concentrations up to 0.4% of glutaraldehyde for immobilization procedures cause a significant decrease of papain activity [35]. Martins et al. [17] working with trehalase of* Escherichia coli *reached a maximum value of 0.026% of enzyme even with the treatment of glutaraldehyde. Therefore, each enzyme may have different sensitivity depending on the support immobilization and concentration of glutaraldehyde. If lesser concentrations of glutaraldehyde were evaluated (e.g., less than 0.1%), maybe the concentration of active papain linkage on chitin would improve, due to less inhibition of the catalytic site of this enzyme and better protein immobilization, probably improving the yield of active enzyme immobilization.

#### 3.3.2. Immobilization on Chitosan

The highest activities of papain immobilized on chitosan were in protocols A′ and B′ both in 5 h, respectively, 2.07 U/g and 1.24 U/g support (Tables 6 and 7). The yield of active papain immobilized on chitosan (Table 7) cross-linked with 1% TPP (protocol B′) was higher (15.7%) than in protocol B on chitin (6.07%) with 0.1% PEI, both at the same time (5 h). In protocol B′ the high concentration of active enzymes in the enzyme solution after immobilization process (86.13% of the total activity) was evident, which could be used for additional immobilization.

Despite the best result of protocol B′ (15.7%), Qiuhua et al. [37] obtained 50% of recovered activity of papain immobilized on microcrystalline chitosan when treated with 3% glutaraldehyde and Hong et al. [38] obtained 66.6%. These values were higher than those found in the present study probably due to the quality of papain and its resistance to glutaraldehyde. The purity of papain could influence the reactions during the immobilization process.

The yields of immobilized protein in protocols A′ and B′ were, respectively, 35% and 9.06% or 24.6 mg and 4.5 mg of protein·g^−1^ of support (Table 7). The time of immobilization process is also important. The yield of immobilized protein in protocol B′ in 5 h and 12 h was, respectively, 9.06% and 45.9%. However if the time of immobilization is important to increase the yield of immobilized protein, the yield of active immobilized enzyme decreased with the time, from 15.73% in 5 h to only 1.94% in 12 h (Table 7). Therefore, the time of enzyme immobilization is critical and more studies are justified in order to improve this process.

In relation to the yield of active immobilized papain, Lorenzoni et al. [18] worked with fructooligosaccharides and inverted sugar by *β*-fructofuranosidase and *β*-fructosyltransferase in a covalent immobilization on chitosan spheres, using glutaraldehyde as a coupling agent. 42% of immobilization yield and 12% of immobilization efficiency (yield of active immobilized enzyme) were obtained in these conditions. This yield was lower than that obtained with the immobilization of papain on chitosan and TPP in the present work (15.7%).

The best yields of immobilized protein (Table 7) were, respectively, for protocols A′, B′, and C′ (44–46.6%) in 12 h of reaction. These are high yields if they are compared with the literature. Spagna et al. [20] working with *α*-L-arabinofuranosidase immobilized on chitosan obtained a lesser yield from 25% to 30%. Zappino et al. [39] working with immobilization of bromelain on microbial and animal chitosan treated with 25% of glycerol achieved the best result of 41% of protein immobilization yield in films of chitosan. In another study with *β*-galactosidase immobilized on chitosan, 60% of protein immobilization yield on support of alginate-chitosan was performed [40].

However, the yield of immobilized protein is not an efficient parameter to evaluate the immobilization of enzyme. The best protocols for protein immobilization (A′, B′, and C′) in 12 h reaction showed lower yield of immobilized active papain when compared with the same protocols in 5 h of reaction (Table 7). Probably the catalytic site of this enzyme could be inactivated by enzyme denaturation due to higher time of contact between papain and 1% TPP, since in the test of evaluation of TPP concentration on the activity of soluble papain there was a decrease in the enzyme activity from 2.61 to 1.43 U/mL with the increase of TPP from 0 to 1.0% (Table 5).

Protocol B′ showed the best yield of active immobilized enzyme, but the yield of active immobilized protein was only 9.06% (Table 7). However, this result was higher than that found by Hong et al. [38] also working with papain immobilization on chitosan microspheres, cross-linked with 0.5% glutaraldehyde (0.19%).

### 3.4. Yeast Deflocculation Using Immobilized Papain on Chitin and Chitosan

The immobilized papain was tested in flocculated* S. cerevisiae* suspension from ethanol distillery in 120 min of reaction using 2 g of support (chitin or chitosan) in 50 mL yeast suspension. In the case of chitin protocol B was selected (Tables 1 and 7) and for chitosan protocol B′ (Tables 2 and 7).

**Figure 2:**
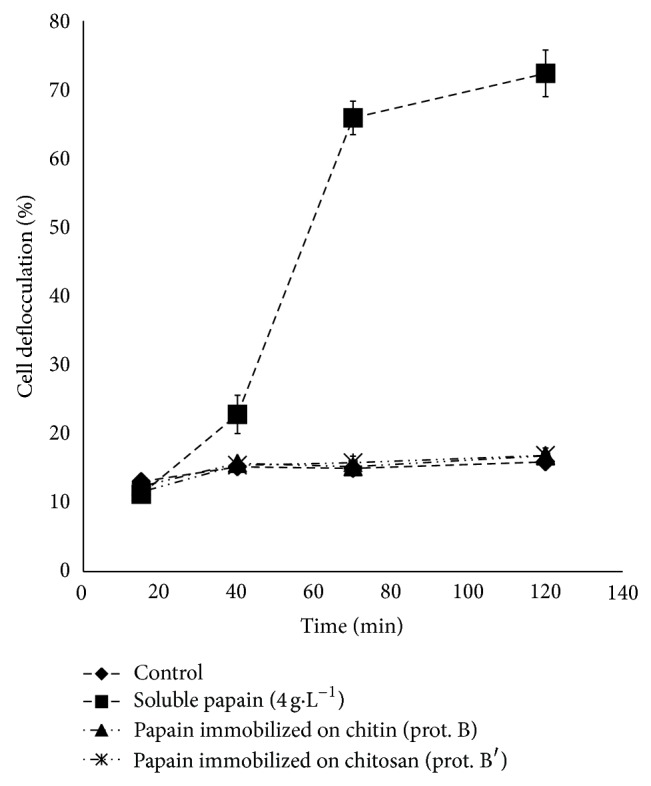
Effect of the soluble and immobilized papain in the suspension of flocculated yeast from fuel ethanol distillery.

Yeast suspension treated with the immobilized enzymes showed no significant differences (*P* < 0.05) when compared to the control (flocculated). After 120 minutes of reaction, both treatments with the immobilized enzyme showed only 15% of deflocculation, while the yeast treated with 4 g·L^−1^ soluble papain showed more than 70% of yeast cells deflocculation (Figure 2). These results were expected since there was a relatively low concentration of active enzyme in both supports, since for chitosan (protocol B′) there was a yield of active immobilized enzyme of 15.7% and for chitin (protocol B) only 6.07% (Table 7).

The ideal concentration of active papain in immobilized support for yeast deflocculation can be estimated. If 10 g·L^−1^ papain solution showed 2.04–2.68 U/mL (Table 5), there is 0.200–0.268 U/mg papain. If 4 g·L^−1^ of papain in 50 mL of yeast suspension was necessary to obtain 70% deflocculation of yeast cells, it means 40 U to 53.6 U or 46.8 U in average of total proteolytic activity was needed to deflocculate 50 mL of yeast suspension. 2 g of papain immobilized on chitin or chitosan was used in yeast deflocculation tests, with, respectively, 0.11 U/g (protocol B) and 1.24 U/g (protocol B′) (Table 7). Therefore, 0.22 U for chitin or 2.48 U for chitosan of total papain activity was used in 50 mL of 30% yeast suspension. These values were not enough for yeast deflocculation, since they represent only 0.47% (for chitin) and 5.3% (for chitosan) of the total protease activity spent in the deflocculation tests with soluble enzymes.

The yeast deflocculation may be possible if papain immobilized in these supports was packaged in columns of a plug flow reactor and the flocculated yeast suspension was carried through the reactor. Using this process there will be an increase in the percentage of active immobilized papain to yeast cells deflocculation, but this condition needs to be tested.

The purity of enzyme is another important point to improve the efficiency in yield of immobilization. The higher the purity of the enzyme obtained the better the efficiency in the enzyme immobilization. In this work only 3.6% of protein was determined in the papain used for immobilization tests. In addition, the improvement in the concentration of the active enzyme in derivatives for yeast cell deflocculation is also possible by other pretreatments of supports, the use of more resistant protease for cross-linking agent, and other techniques as multipoint covalent immobilization of enzymes [41].

### 3.5. Reuse of the Soluble Papain by Centrifugation

**Figure 3:**
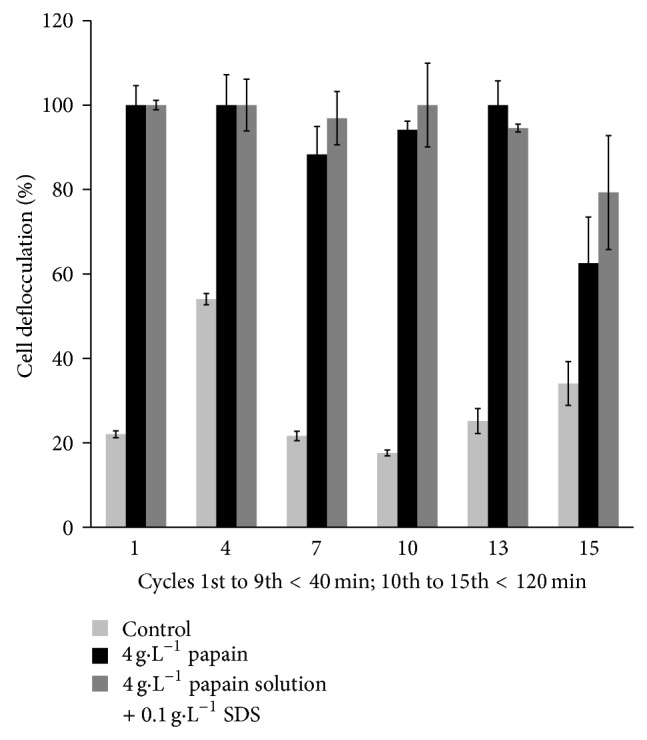
Yeast cell deflocculation with the recycle of soluble papain by centrifugation of yeast suspension and enzyme recovery.

The flocculated* S. cerevisiae *suspension was reacted with a papain solution and SDS in 15 to 120 min (Figure 3) of reaction time. The results were very interesting since yeast deflocculation was possible in a short time and at the temperature of the industrial process (25–30°C). 14 recycles of papain on yeast cells deflocculation was possible with this proposed method. In addition, just only 5% of new enzyme solution after each cycle of deflocculation was needed to add on the yeast cell suspension.

In the treatment using soluble papain (4 g·L^−1^) and 0.10 g·L^−1^ of SDS for yeast deflocculation a slightly better performance of SDS was observed when compared to the treatment with soluble papain (4 g·L^−1^). However, there was no significant difference (*P* < 0.05) in these treatments, therefore not justifying the use of SDS in cell deflocculation by soluble papain (Figure 3). The use of low concentrations of SDS in this work is justified by the toxicity of this product to yeast metabolism in concentrations higher than 0.30 g·L^−1^, as observed by de Oliva-Neto and Yokoya [10].

The acid treatment could be excessively abrasive on the yeast wall, which is essential for cell viability and production of ethanol, and according to Paterson et al. [42] the intensity of the acid treatment changes with the level of contamination and flocculation. On the other hand, the use of sulfuric acid as the agent for disinfecting the yeast cream and yeast deflocculation constitutes one of the most efficient and economic practices to the production of fuel ethanol. However, it should not be indiscriminately used as it currently is but carefully monitored during fermentation process [43].

Although some researchers advocate the use of sulfuric acid to decontaminate the alcoholic fermentation process, for others this practice is not efficient, raising the cost of ethanol due to the increase in the use of this product and damaging the environment since the acid vinasse is placed in the soil [44].

An alternative treatment with the use of enzymes has been proposed by Ludwig [13] with two enzymes, protease (Novozyme 642) and carbohydrase (Novozyme SP 299), which have been proved to be effective in yeast deflocculation and further they did not affect the yeast viability. However, these enzymes are not economically viable for industrial application due to the need for high dosages. Therefore, the development of technology responsible for cost reduction of enzymes in specific applications could contribute to become economically viable the use of these biocatalysts in the industry.

Currently, the yeast cells are centrifuged one time in fed-batch or continuous process with cell recycle in Brazilian distilleries [2]. The concept of this purposed new method is to improve the industrial process of fuel ethanol technology with the removal of acid treatment of yeast cells or its reduction replacing the acid by protease treatment in a new double yeast centrifugation. Firstly, yeast suspension (after the first centrifugation) will be treated with soluble proteases for a certain time (15–120 min). After yeast deflocculation, the yeast cells will be separated by the second centrifugation and enzyme solution will be recovered to be used in another treatment with another yeast suspension. The proposed method was demonstrated to be effective at yeast deflocculation in 14 recycles of papain. However, a test on an industrial scale is necessary to prove the efficiency of this method. After an economic analysis, maybe a new alternative to the acid treatment of yeast will be possible, especially when the yeast viability is low and the acid treatment is not recommended.

## 4. Conclusion

The inhibitory effect of some cross-linking agents to papain activity was evaluated. The glutaraldehyde and polyethyleneimine in concentrations, respectively, 0.10% and 1%, strongly inhibited the activity of soluble papain. However, this enzyme was partly inhibited by tripolyphosphate (TPP) at the minimum concentration required for cross-linking with chitosan. The treatment of soluble papain in* S. cerevisiae* cells was efficient in yeast cell deflocculation in a short time, maintaining yeast cell viability, but only in a high dosage. This method could replace the current method used in industry with sulfuric acid, but the reuse of the enzyme is needed for an economical process on an industrial scale.

Several protocols of papain immobilization were performed. The protocols with papain on chitin and glutaraldehyde had a higher protein immobilization, but the great inhibition of papain by this agent was confirmed. Chitosan cross-linked with tripolyphosphate showed higher yield of active immobilized enzyme than chitin treated with polyethyleneimine. Although these immobilizations have been possible, these levels have not been enough to cause yeast cells deflocculation in a reaction by batch process; therefore higher active papain pellets are required.

Recycling of free enzyme by centrifugation was effective for 14 cycles with yeast suspension in a time perfectly compatible to industrial conditions. The reuse of proteases applied in yeast suspension by additional yeast centrifugation could be a new alternative to sulfuric acid, especially when the yeast viability is low and the treatment with sulfuric acid is not recommended.

## Supplementary Material

Mechanism of yeast and bacteria cells flocculation: The flocculation mechanism between cells of contaminating bacteria and yeasts in fuel ethanol fermentation is associated with the physical contact between the cell wall of these microorganisms. There is an optimum relationship between the amount of bacteria and yeast cells needed to cause cells flocculation. According Yokoya and Oliva-Neto [7] by microscopic count this ratio is 4.8 (bacteria/yeast), which explains the sudden appearance of yeast flocculation in fuel ethanol production industries, when there is an increase in bacterial contamination by mainly *Lactobacillus fermentum*. Amino acid residues on surface of *Lactobacillus fermentum* cell wall and carbohydrate residues on yeast cell wall are responsible for the development of yeast cells flocculation. Some reactions with specific reagent which modified proteins indicated the indole group of tryptophan and phenolic hydroxyl group of tyrosine must be present on the bacterial cell surface for the flocculation to occur. The residues of mannan on the cell surface of yeast are responsible for this binding [6]. Therefore, protein factors associated with minerals such as Ca^+2^ [5], as well as mannans, have been proved to be involved in the process of flocculation.

## Figures and Tables

**Table 1:** Protocols used for papain immobilized on chitin.

Treatment	Immobilization conditions	Pretreatment of support
A	1 g chitin + 1% papain^a^	—
B	1 g chitin + 1% papain + 0.1% PEI	—
C	1 g chitin + 1% papain + 0.5% PEI	—
D	1 g chitin + 1% papain	2% glutaraldehyde^b^
E	1 g chitin + 1% papain + 0.1% PEI^c^	2% glutaraldehyde
F	1 g chitin + 1% papain + 0.5% PEI	2% glutaraldehyde

^a^10 mL of 10 g·L^−1^; ^b^10 mL of 2% (v·v^−1^) glutaraldehyde; ^c^0.1 or 0.5% (v·v^−1^) in papain solution.

**Table 2:** Protocols used for papain immobilized on chitosan.

Treatment	Preparation of chitosan with TPP	Adsorption of papain on chitosan with TPP	Reaction time (h)
A′	1 g chitosan in 100 mL 1% acetic acid solution dripped into 200 mL 1% TPP	Washing and addition of the microspheres in 200 mL 1% papain solution (phosphate buffer pH 6.4)	5 and 12

B′	1 g chitosan in 100 mL 1% acetic acid solution dripped into a 200 mL 1% TPP and 1% papain to produce microspheres	—	5 and 12

C′	1 g chitosan in 100 mL 1% acetic acid solution dripped into a solution of 200 mL 1% TPP	Addition of the 1% papain solution instantly after the formation of microspheres	5 and 12

**Table 3:** Percentage of *S.  cerevisiae* cells deflocculation, yeast precipitation, and turbidity measured in 600 nm by spectrophotometry.

Total turbidity (600 nm)	Yeast deflocculation (%)	Yeast precipitation
≤12.0	0	p^2^
27.0	25.0	np^3^
40.5	37.5	np
54.0	50.0	np
67.5	62.5	np
81.0	75.0	np
94.5	87.5	np
≥120.0^1^	100	np

^1^Maximum deflocculation of 30% (w·v^−1^) yeast cell solution by sulfuric acid; ^2^p = cells precipitated in the bottom of the suspension; ^3^np = cells suspended in liquid.

**Table 4:** Viability of yeast cell treated with H_2_SO_4_ and papain in 2 h of reaction.

Treatments	Cell viability (%)
Control	72.0 ± 1.0^a^
Sulfuric acid^1^	67.19 ± 0.8^b^
Papain^2^	72.4 ± 1.4^a^

^1^Concentration to induce yeast cells deflocculation—pH 2.5; ^2^4 g·L^−1^; different letters indicate that they are statistically different (*P* < 0.05).

**Table 5:** Proteolytic activity of the papain in the presence of glutaraldehyde, polyethyleneimine, and tripolyphosphate, for 120 minutes at 27°C.

Concentration (%)	Glutaraldehyde (U/mL)	Polyethyleneimine (PEI) (U/mL)	Sodium tripolyphosphate (U/mL)
Control	2.04 ± 0.04^a^	2.68 ± 0.09^a^	2.61 ± 0.07^a^
0.1	0.18 ± 0.06^b^	3.01 ± 0.04^b^	1.68 ± 0.09^b^
0.5	0.13 ± 0.01^b^	2.25 ± 0.24^c^	1.47 ± 0.10^c^
1.0	0.12 ± 0.00^b^	0.96 ± 0.07^d^	1.43 ± 0.09^c^
1.5	0.12 ± 0.02^b^	0.81 ± 0.08^d^	0.88 ± 0.10^d^
2.0	0.11 ± 0.02^b^	0.58 ± 0.06^d^	0.55 ± 0.04^e^
5.0	0.01 ± 0.01^c^	0.74 ± 0.14^d^	0.48 ± 0.09^e^
10.0	0.00 ± 0.00^c^	0.59 ± 0.27^d^	0.34 ± 0.00^e^

Obs. different letters indicate that they are statistically different (*P* < 0.05). Papain concentration was 10 g·L^−1^ (1%—w·v^−1^).

**Table 6:** Total activity of papain in several steps, according to each protocol of immobilization.

Immobilization protocol^a^	Total enzyme in solution	Enzyme solution after the immobilization process	1st pellets washing-water (100 mL)	2nd pellets washing-water (1000 mL)	Active immob. enzyme (U)^c^
A	355.14	215.88 (14)^b^	74.0	0.0	1.53 (6.5)^d^
B	481.34	333.48 (14)	136.0	0.0	0.72 (6.5)
C	416.86	122.46 (13)	148.0	66.7	0.43 (6.5)
D	316.00	132.66 (18)	48.0	0.0	0.00 (7.5)
E	360.94	240.79 (18)	70.3	31.0	0.00 (7.5)
F	424.46	225.54 (18)	87.0	0.0	0.10 (7.5)
A′-5 h	206.50	55.97 (39)	0.0	0.0	5.76 (2.78)
B′-5 h	180.65	155.61 (26)	0.0	0.0	3.94 (3.17)
C′-5 h	201.00	138.13 (33)	0.0	0.0	2.24 (2.70)
A′-12 h	184.75	34.59 (30)	0.0	0.0	2.21 (2.66)
B′-12 h	197.50	6.65 (25)	0.0	0.0	3.72 (2.86)
C′-12 h	197.75	29.92 (34)	0.0	0.0	2.34 (2.84)

^a^Protocols in Tables 1 and 2; ^b^remaining volume (mL) obtained from each immobilization is indicated in parentheses; ^c^total units of immobilized enzyme, and ^d^wet weight (g) of pellets.

**Table 7:** Determination of yield of active immobilized papain and other parameters.

Immobilization protocols^a^	Enzyme activity after immobilization (in solution) (%)	Enzyme activity involved in immobilization (%)	Yield^c^ of active immobilized enzyme (%)	Yield^d^of immobilized protein (%) and immob. protein on support (mg·g^−1^)	Enzymatic activity in support (U/g)
A	81.62	18.37	2.34	0.24 (0.12)	0.23
B	97.53	2.46	6.07	0.3 (0.148)	0.11
C	64.88	35.11	0.29	0.3 (0.15)	0.06
D	57.17	42.82	0.00	13.56 (6.73)	0.00
E	86.18	13.81	0.00	43.58 (21.63)	0.00
F	73.63	26.36	0.08	67.41 (33.46)	0.013
A′-5 h	27.10	72.89	3.82	35.00 (24.60)	2.07
B′-5 h	86.13	13.86	15.73	9.06 (4.50)	1.24
C′-5 h	68.72	31.27	3.56	45.9 (23.90)	0.82
A′-12 h	18.72	81.27	1.47	44.06 (30.80)	0.83
B′-12 h	3.36	96.63	1.94	45.9 (23.90)	1.30
C′-12 h	15.13	84.86	1.39	46.64 (29.40)	0.82

^a^Protocols in Tables 1 and 2; ^b^calculated as *B* × *A* ^−1^  × 100 where *B* is the total activity of the enzyme remaining in solution after immobilization process; *A* is the total activity of the enzyme added to the support; ^c^calculated as total activity of immobilized enzyme (U) × (*A* − *B*)^−1^ × 100; ^d^calculated as *C* × *D* ^−1^  × 100 where *C* is the immobilized protein (g); *D* is total protein added on support (g), total protein remaining in solution after the immobilization process (g).

## References

[1] Ludwig K. M., Oliva-Neto P., Angelis D. F. (2001). Quantification of *Saccharomyces cerevisiae* flocculation by bacterial contaminants of alcoholic fermentation. *Ciência e Tecnologia Alimentos*.

[2] de Oliva-Neto P., Yokoya F. (1994). Evaluation of bacterial contamination in a fed-batch alcoholic fermentation process. *World Journal of Microbiology and Biotechnology*.

[3] Oliva-Neto P., Dorta C., Carvalho A. F. A., Lima V. M. G., Silva D. F., Méndez-Vilas A. (2013). The Brazilian technology of fuel ethanol fermentation—yeast inhibition factors and new perspectives to improve the technology. *Materials and Processes for Energy: Communicating Current Research and Technological Developments*.

[4] Migneault I., Dartiguenave C., Bertrand M. J., Waldron K. C. (2004). Glutaraldehyde: behavior in aqueous solution, reaction with proteins, and application to enzyme crosslinking. *BioTechniques*.

[5] Oliva Neto P., Ludwig K. M., Dorta C., Carvalho A. F. A., Silva D. F., Lima V. M. G., Stradioto N., Lemos E. (2012). Microbial contamination of the alcholic fermentation for fuel ethanol production. *Bioenergy: Developing, Research and Inovation*.

[6] Santos M. T., Yokoya F. (1993). Characteristics of yeast cell flocculation by *Lactobacillus fermentum*. *Journal of Fermentation and Bioengineering*.

[7] Yokoya F., Oliva-Neto P. (1991). Characterization of yeast flocculation by *Lactobacillus fermentum*. *Revista de Microbiologia*.

[8] Hynes S. H., Kjarsgaard D. M., Thomas K. C., Ingledew W. M. (1997). Use of virginiamycin to control the growth of lactic acid bacteria during alcohol fermentation. *Journal of Industrial Microbiology and Biotechnology*.

[9] Meneghin S. P., Reis F. C., De Almeida P. G., Ceccato-Antonini S. R. (2008). Chlorine dioxide against bacteria and yeasts from the alcoholic fermentation. *Brazilian Journal of Microbiology*.

[10] de Oliva-Neto P., Yokoya F. (1998). Effect of 3,4,4′-trichlorocarbanilide on growth of lactic acid bacteria contaminants in alcoholic fermentation. *Bioresource Technology*.

[11] Dorta C., Oliva-Neto P., de-Abreu-Neto M. S., Nicolau-Junior N., Nagashima A. I. (2006). Synergism among lactic acid, sulfite, pH and ethanol in alcoholic fermentation of *Saccharomyces cerevisiae* (PE-2 and M-26). *World Journal of Microbiology and Biotechnology*.

[12] Azarkan M., El Moussaoui A., van Wuytswinkel D., Dehon G., Looze Y. (2003). Fractionation and purification of the enzymes stored in the latex of *Carica papaya*. *Journal of Chromatography B: Analytical Technologies in the Biomedical and Life Sciences*.

[13] Ludwig K. M. (1998). *Flocculation of Saccharomyces cerevisiae—characterization and the action of deflocculation enzymes [Master in Microbiology]*.

[14] Luque S., Benito J. M., Coca J. (2004). The importance of specification sheets for pressure-driven membrane processes. *Filtration and Separation*.

[15] de Oliva-Neto P., Menão P. T. P. (2009). Isomaltulose production from sucrose by *Protaminobacter rubrum* immobilized in calcium alginate. *Bioresource Technology*.

[16] Kubota N., Tatsumoto N., Sano T., Toya K. (2000). A simple preparation of half N-acetylated chitosan highly soluble in water and aqueous organic solvents. *Carbohydrate Research*.

[17] Martins A. S., Peixoto D. N., Paiva L. M. C., Panek A. D., Paiva C. L. A. (2006). A simple method for obtaining reusable reactors containing immobilized trehalase: characterization of a crude trehalase preparation immobilized on chitin particles. *Enzyme and Microbial Technology*.

[18] Lorenzoni A. S. G., Aydos L. F., Klein M. P., Ayub M. A. Z., Rodrigues R. C., Hertz P. F. (2014). Continuous production of fructooligosaccharides and invert sugar by chitosan immobilized enzymes: comparison between in fluidized and packed bed reactors. *Journal of Molecular Catalysis B: Enzymatic*.

[19] Chen H., Zhang Q., Dang Y., Shu G. (2013). The effect of glutaraldehyde cross-linking on the enzyme activity of immobilized *β*-galactosidase on chitosan bead. *Advance Journal of Food Science and Technology*.

[20] Spagna G., Andreani F., Salatelli E., Romagnoli D., Pifferi P. G. (1998). Immobilization of *α*-L-arabinofuranosidase on chitin and chitosan. *Process Biochemistry*.

[21] Anthonsen M. W., Vårum K. M., Smidsrød O. (1993). Solution properties of chitosans: conformation and chain stiffness of chitosans with different degrees of *N*-acetylation. *Carbohydrate Polymers*.

[22] Krajewska B. (1991). Chitin and its derivatives as supports for immobilization of enzymes. *Acta Biotechnologica*.

[23] Laus R., Laranjeira M. C. M., Martins A. O. (2006). Chitosan microspheres crosslinked with tripolyphosphate used for the removal of the acidity, iron (III) and manganese (II) in water contaminated in coal mining. *Química Nova*.

[24] Koudelka-Hep M., Rooij N. F., Strike D. J., Bickerstaff F. G. (1997). Immobilization of enzymes on microelectrodes using chemical crosslinking. *Immobilization of Enzimes and Cells*.

[25] Broun G. B. (1976). Chemically aggregated enzymes. *Methods in Enzymology*.

[26] Dalla-Vecchia R., Nascimento M. D. G., Soldi V. (2004). Synthetic applications of immobilized lipases in polymers. *Química Nova*.

[27] Leighton T. J., Doi R. H., Warren R. A. J., Kelln R. A. (1973). The relationship of serine protease activity to RNA polymerase modification and sporulation in *Bacillus subtilis*. *Journal of Molecular Biology*.

[28] Bradford M. M. (1976). A rapid and sensitive method for the quantitation of microgram quantities of protein utilizing the principle of protein-dye binding. *Analytical Biochemistry*.

[29] Varavinit S., Chaokasem N., Shobsngob S. (2002). Immobilization of a thermostable alpha-amylase. *Science Asia*.

[30] Johnstone A., Thorpe R. (1987). *Immunochemistry in Practice*.

[31] Vasconcellos F. C., Goulart G. A. S., Beppu M. M. (2011). Production and characterization of chitosan microparticles containing papain for controlled release applications. *Powder Technology*.

[32] Lima R. S., Nunes G. S., Noguer T., Marty J.-L. (2007). Enzymatic biosensor for the detection of dithiocarbamate fungicides. Kinetic study of aldehyde dehydrogenase enzyme and biosensor optimization. *Química Nova*.

[33] Zaborsky O. (1973). *Immobilized Enzymes*.

[34] Chui W. K., Wan L. S. C. (1997). Prolonged retention of cross-linked trypsin in calcium alginate microspheres. *Journal of Microencapsulation*.

[35] Li Y. F., Jia F. Y., Li J. R., Liu G., Li Y. Z. (2001). Papain immobilization on a nitrilon fibre carrier containing primary amine groups. *Biotechnology and Applied Biochemistry*.

[36] Gaspari J. W., Gomes L. H., Tavares F. C. A. (1999). Immobilization of inulinase from *Kluyveromyces marxianus* for the hydrolysis of extracts of *Helianthus tuberosus* L. *Scientia Agricola*.

[37] Qiuhua L., Jingesu M., Gangjun G., Xu C., Hong X. (1998). Immobilization of Papain on microcrystalline chitosan. *Journal of Branch Campus of the First Military Medical*.

[38] Hong L., Jun W. W., Cai X. F. (2000). Studies on preparation of chitosan microspheres and immobilization of papain. *Journal of South China Agricultural University*.

[39] Zappino M., Cacciotti I., Benucci I. (2015). Bromelain immobilization on microbial and animal source chitosan films, plasticized with glycerol, for application in wine-like medium: microstructural, mechanical and catalytic characterisations. *Food Hydrocolloids*.

[40] Taqieddin E., Amiji M. (2004). Enzyme immobilization in novel alginate-chitosan core-shell microcapsules. *Biomaterials*.

[41] Guisán J. M., Bastida A., Blanco R. M., Fernández-Lafuente R., García-Junceda E., Bickerstaff G. F. (1997). Immobilization of enzymes on glyoxyl agarose. *Immobilization of Enzymes and Cells*.

[42] Paterson M., Borba J. M. M., Melo F. A. D., Moraes J. I. (1988). Evaluating the performance of ethanol fermentation in different situations of industrial process. *Brasil Açucareiro*.

[43] Alves da Silva F. E. (1993). *Ethanolic fermentation: influence of sulfuric acid on yeast viability and bacterial and yeast contaminants [M.S. thesis]*.

[44] Otênio M. H. (1998). *Comparative evaluation of the effect of removal of acid treatment with sulfuric acid in yeast during the recycles in the Bandeirantes Distillery (Brazil) [Master degree]*.

