# Supplementary material for: Immobilization of Papain on Chitin and Chitosan and Recycling of Soluble Enzyme for Deflocculation of Saccharomyces cerevisiae from Bioethanol Distilleries
Source: Enzyme Res. 2015 Jan 1;2015:573721. doi: 10.1155/2015/573721 (PMC4299301; doi:10.1155/2015/573721)
Supplement: Supplementary file 1 — Mechanism of yeast and bacteria cells flocculation: The flocculation mechanism between cells of contaminating bacteria and yeasts in fuel ethanol fermentation is associated with the physical contact between the cell wall of these microorganisms. There is an optimum relationship between the amount of bacteria and yeast cells needed to cause cells flocculation. According Yokoya and Oliva-Neto [7] by microscopic count this ratio is 4.8 (bacteria/yeast), which explains the sudden appearance of yeast flocculation in fuel ethanol production industries, when there is an increase in bacterial contamination by mainly Lactobacillus fermentum. Amino acid residues on surface of Lactobacillus fermentum cell wall and carbohydrate residues on yeast cell wall are responsible for the development of yeast cells flocculation. Some reactions with specific reagent which modified proteins indicated the indole group of tryptophan and phenolic hydroxyl group of tyrosine must be present on the bacterial cell surface for the flocculation to occur. The residues of mannan on the cell surface of yeast are responsible for this binding [6]. Therefore, protein factors associated with minerals such as Ca+2 [5], as well as mannans, have been proved to be involved in the process of flocculation. [file 573721.f1.pdf]

Appendix 1

Fig. 1 Statistic - Means

Comparison	Difference	q	P value
control vs 1mg/mL	-21.253	9.238	*** P<0.001
control vs 3mg/mL	-48.810	21.217	*** P<0.001
control vs 4mg/mL	-60.410	26.260	*** P<0.001
1mg/mL vs 3mg/mL	-27.557	11.979	*** P<0.001
1mg/mL vs 4mg/mL	-39.157	17.021	*** P<0.001
3mg/mL vs 4mg/mL	-11.600	5.042	* P<0.05

Difference	Mean Difference	95% Confidence Interval From	To
control - 1mg/mL	-21.253	-31.672	-10.834
control - 3mg/mL	-48.810	-59.229	-38.391
control - 4mg/mL	-60.410	-70.829	-49.991
1mg/mL - 3mg/mL	-27.557	-37.976	-17.138
1mg/mL - 4mg/mL	-39.157	-49.576	-28.738
3mg/mL - 4mg/mL	-11.600	-22.019	-1.181

Intermediate calculations. ANOVA table

Source of variation	Degrees of freedom	Sum of squares	Mean square
Treatments (between columns)	3	6683.0	2227.7
Residuals (within columns)	8	127.01	15.877
Total	11	6810.0	

F = 140.31 =(MStreatment/MSresidual)

Appendix 2

Fig. 2 Statistic

Table for statistic figure 2: Immobilization A (chitin), B' (chitosan) and Soluble papain (4 g x L^{-1}) in process of deflocculation (30% flocculated yeast - *w x v^{-1}*) for 15, 40, 70 and 120 minutes compared with control (p<0.05).

	**Protocol A**				**Protocol B'**				**Soluble Papain (4 g x L^{-1})**			
**Control**	15'	40'	70'	120'	15'	40'	70'	120'	15'	40'	70'	120'
15'	ns				ns				s			
40'		ns				ns				s		
70'			ns				ns				s	
120'				ns				ns				s

ns – no signifcative (p>0.05), s signifcative (p<0.05).

```
                    Mean
Comparison          Difference   q      P value
================================ ========= ======= ===========
control 15' vs control 40'       -2.157   2.574  ns  P>0.05
control 15' vs control 70'       -1.926   2.298  ns  P>0.05
control 15' vs control 120'      -2.876   3.432  ns  P>0.05
control 15' vs 4mg/ml 15'         1.815   2.166  ns  P>0.05
control 15' vs 4mg/mL 40'        -9.769  11.658 *** P<0.001
control 15' vs 4mg/mL 70'       -52.852  63.073 *** P<0.001
control 15' vs 4mg/mL 120'      -59.315  70.786 *** P<0.001
control 15' vs Protocol A 15'    0.6940  0.8282  ns  P>0.05
control 15' vs Protocol A 40'    -2.667   3.183  ns  P>0.05
control 15' vs Protocol A 70'    -2.176   2.597  ns  P>0.05
control 15' vs Protocol A 120'   -3.759   4.486  ns  P>0.05
control 15' vs Protocol B' 15'    1.500   1.790  ns  P>0.05
control 15' vs Protocol B'40'    -2.389   2.851  ns  P>0.05
control 15' vs Protocol B' 70'   -2.750   3.282  ns  P>0.05
control 15' vs Protocol B' 120   -3.815   4.553  ns  P>0.05
```

control 40' vs control 70'	0.2310	0.2757	ns	P>0.05
control 40' vs control 120'	-0.7190	0.8580	ns	P>0.05
control 40' vs 4mg/ml 15'	3.972	4.740	ns	P>0.05
control 40' vs 4mg/mL 40'	-7.612	9.084	***	P<0.001
control 40' vs 4mg/mL 70'	-50.695	60.499	***	P<0.001
control 40' vs 4mg/mL 120'	-57.158	68.212	***	P<0.001
control 40' vs Protocol A 15'	2.851	3.402	ns	P>0.05
control 40' vs Protocol A 40'	-0.5100	0.6086	ns	P>0.05
control 40' vs Protocol A 70'	-0.01900	0.02267	ns	P>0.05
control 40' vs Protocol A 120'	-1.602	1.912	ns	P>0.05
control 40' vs Protocol B' 15'	3.657	4.364	ns	P>0.05
control 40' vs Protocol B'40'	-0.2320	0.2769	ns	P>0.05
control 40' vs Protocol B' 70'	-0.5930	0.7077	ns	P>0.05
control 40' vs Protocol B' 120	-1.658	1.979	ns	P>0.05
control 70' vs control 120'	-0.9500	1.134	ns	P>0.05
control 70' vs 4mg/ml 15'	3.741	4.464	ns	P>0.05
control 70' vs 4mg/mL 40'	-7.843	9.360	***	P<0.001
control 70' vs 4mg/mL 70'	-50.926	60.775	***	P<0.001
control 70' vs 4mg/mL 120'	-57.389	68.487	***	P<0.001
control 70' vs Protocol A 15'	2.620	3.127	ns	P>0.05
control 70' vs Protocol A 40'	-0.7410	0.8843	ns	P>0.05
control 70' vs Protocol A 70'	-0.2500	0.2983	ns	P>0.05
control 70' vs Protocol A 120'	-1.833	2.187	ns	P>0.05
control 70' vs Protocol B' 15'	3.426	4.089	ns	P>0.05
control 70' vs Protocol B'40'	-0.4630	0.5525	ns	P>0.05
control 70' vs Protocol B' 70'	-0.8240	0.9834	ns	P>0.05
control 70' vs Protocol B' 120	-1.889	2.254	ns	P>0.05
control 120' vs 4mg/ml 15'	4.691	5.598	*	P<0.05
control 120' vs 4mg/mL 40'	-6.893	8.226	***	P<0.001
control 120' vs 4mg/mL 70'	-49.976	59.641	***	P<0.001

control 120' vs 4mg/mL 120'	-56.439	67.354	***	P<0.001
control 120' vs Protocol A 15'	3.570	4.260	ns	P>0.05
control 120' vs Protocol A 40'	0.2090	0.2494	ns	P>0.05
control 120' vs Protocol A 70'	0.7000	0.8354	ns	P>0.05
control 120' vs Protocol A 120'	-0.8830	1.054	ns	P>0.05
control 120' vs Protocol B' 15'	4.376	5.222	ns	P>0.05
control 120' vs Protocol B'40'	0.4870	0.5812	ns	P>0.05
control 120' vs Protocol B' 70'	0.1260	0.1504	ns	P>0.05
control 120' vs Protocol B' 120	-0.9390	1.121	ns	P>0.05
4mg/ml 15' vs 4mg/mL 40'	-11.584	13.824	***	P<0.001
4mg/ml 15' vs 4mg/mL 70'	-54.667	65.239	***	P<0.001
4mg/ml 15' vs 4mg/mL 120'	-61.130	72.952	***	P<0.001
4mg/ml 15' vs Protocol A 15'	-1.121	1.338	ns	P>0.05
4mg/ml 15' vs Protocol A 40'	-4.482	5.349	*	P<0.05
4mg/ml 15' vs Protocol A 70'	-3.991	4.763	ns	P>0.05
4mg/ml 15' vs Protocol A 120'	-5.574	6.652	**	P<0.01
4mg/ml 15' vs Protocol B' 15'	-0.3150	0.3759	ns	P>0.05
4mg/ml 15' vs Protocol B'40'	-4.204	5.017	ns	P>0.05
4mg/ml 15' vs Protocol B' 70'	-4.565	5.448	*	P<0.05
4mg/ml 15' vs Protocol B' 120	-5.630	6.719	**	P<0.01
4mg/mL 40' vs 4mg/mL 70'	-43.083	51.415	***	P<0.001
4mg/mL 40' vs 4mg/mL 120'	-49.546	59.128	***	P<0.001
4mg/mL 40' vs Protocol A 15'	10.463	12.486	***	P<0.001
4mg/mL 40' vs Protocol A 40'	7.102	8.475	***	P<0.001
4mg/mL 40' vs Protocol A 70'	7.593	9.061	***	P<0.001
4mg/mL 40' vs Protocol A 120'	6.010	7.172	**	P<0.01
4mg/mL 40' vs Protocol B' 15'	11.269	13.448	***	P<0.001
4mg/mL 40' vs Protocol B'40'	7.380	8.807	***	P<0.001
4mg/mL 40' vs Protocol B' 70'	7.019	8.376	***	P<0.001
4mg/mL 40' vs Protocol B' 120	5.954	7.105	**	P<0.01

4mg/mL 70' vs 4mg/mL 120'	-6.463	7.713	***	P<0.001
4mg/mL 70' vs Protocol A 15'	53.546	63.901	***	P<0.001
4mg/mL 70' vs Protocol A 40'	50.185	59.890	***	P<0.001
4mg/mL 70' vs Protocol A 70'	50.676	60.476	***	P<0.001
4mg/mL 70' vs Protocol A 120'	49.093	58.587	***	P<0.001
4mg/mL 70' vs Protocol B' 15'	54.352	64.863	***	P<0.001
4mg/mL 70' vs Protocol B'40'	50.463	60.222	***	P<0.001
4mg/mL 70' vs Protocol B' 70'	50.102	59.791	***	P<0.001
4mg/mL 70' vs Protocol B' 120	49.037	58.520	***	P<0.001
4mg/mL 120' vs Protocol A 15'	60.009	71.614	***	P<0.001
4mg/mL 120' vs Protocol A 40'	56.648	67.603	***	P<0.001
4mg/mL 120' vs Protocol A 70'	57.139	68.189	***	P<0.001
4mg/mL 120' vs Protocol A 120'	55.556	66.300	***	P<0.001
4mg/mL 120' vs Protocol B' 15'	60.815	72.576	***	P<0.001
4mg/mL 120' vs Protocol B'40'	56.926	67.935	***	P<0.001
4mg/mL 120' vs Protocol B' 70'	56.565	67.504	***	P<0.001
4mg/mL 120' vs Protocol B' 120	55.500	66.233	***	P<0.001
Protocol A 15' vs Protocol A 40'	-3.361	4.011	ns	P>0.05
Protocol A 15' vs Protocol A 70'	-2.870	3.425	ns	P>0.05
Protocol A 15' vs Protocol A 120'	-4.453	5.314	*	P<0.05
Protocol A 15' vs Protocol B' 15'	0.8060	0.9619	ns	P>0.05
Protocol A 15' vs Protocol B'40'	-3.083	3.679	ns	P>0.05
Protocol A 15' vs Protocol B' 70'	-3.444	4.110	ns	P>0.05
Protocol A 15' vs Protocol B' 120	-4.509	5.381	*	P<0.05
Protocol A 40' vs Protocol A 70'	0.4910	0.5860	ns	P>0.05
Protocol A 40' vs Protocol A 120'	-1.092	1.303	ns	P>0.05
Protocol A 40' vs Protocol B' 15'	4.167	4.973	ns	P>0.05
Protocol A 40' vs Protocol B'40'	0.2780	0.3318	ns	P>0.05
Protocol A 40' vs Protocol B' 70'	-0.08300	0.09905	ns	P>0.05
Protocol A 40' vs Protocol B' 120	-1.148	1.370	ns	P>0.05

Protocol A 70' vs Protocol A 120' -1.583 1.889 ns P>0.05

Protocol A 70' vs Protocol B' 15' 3.676 4.387 ns P>0.05

Protocol A 70' vs Protocol B'40' -0.2130 0.2542 ns P>0.05

Protocol A 70' vs Protocol B' 70' -0.5740 0.6850 ns P>0.05

Protocol A 70' vs Protocol B' 120 -1.639 1.956 ns P>0.05

Protocol A 120' vs Protocol B' 15' 5.259 6.276 ** P<0.01

Protocol A 120' vs Protocol B'40' 1.370 1.635 ns P>0.05

Protocol A 120' vs Protocol B' 70' 1.009 1.204 ns P>0.05

Protocol A 120' vs Protocol B' 120 -0.05600 0.06683 ns P>0.05

Protocol B' 15' vs Protocol B'40' -3.889 4.641 ns P>0.05

Protocol B' 15' vs Protocol B' 70' -4.250 5.072 ns P>0.05

Protocol B' 15' vs Protocol B' 120 -5.315 6.343 ** P<0.01

Protocol B'40' vs Protocol B' 70' -0.3610 0.4308 ns P>0.05

Protocol B'40' vs Protocol B' 120 -1.426 1.702 ns P>0.05

Protocol B' 70' vs Protocol B' 120 -1.065 1.271 ns P>0.05

	Mean	95% Confidence Interval	
Difference	Difference	From	To
control 15' - control 40'	-2.157	-6.556	2.242
control 15' - control 70'	-1.926	-6.325	2.473
control 15' - control 120'	-2.876	-7.275	1.523
control 15' - 4mg/ml 15'	1.815	-2.584	6.214
control 15' - 4mg/mL 40'	-9.769	-14.168	-5.370
control 15' - 4mg/mL 70'	-52.852	-57.251	-48.453
control 15' - 4mg/mL 120'	-59.315	-63.714	-54.916
control 15' - Protocol A 15'	0.6940	-3.705	5.093
control 15' - Protocol A 40'	-2.667	-7.066	1.732
control 15' - Protocol A 70'	-2.176	-6.575	2.223
control 15' - Protocol A 120'	-3.759	-8.158	0.6397

control 15' - Protocol B' 15'	1.500	-2.899	5.899
control 15' - Protocol B'40'	-2.389	-6.788	2.010
control 15' - Protocol B' 70'	-2.750	-7.149	1.649
control 15' - Protocol B' 120	-3.815	-8.214	0.5837
control 40' - control 70'	0.2310	-4.168	4.630
control 40' - control 120'	-0.7190	-5.118	3.680
control 40' - 4mg/ml 15'	3.972	-0.4267	8.371
control 40' - 4mg/mL 40'	-7.612	-12.011	-3.213
control 40' - 4mg/mL 70'	-50.695	-55.094	-46.296
control 40' - 4mg/mL 120'	-57.158	-61.557	-52.759
control 40' - Protocol A 15'	2.851	-1.548	7.250
control 40' - Protocol A 40'	-0.5100	-4.909	3.889
control 40' - Protocol A 70'	-0.01900	-4.418	4.380
control 40' - Protocol A 120'	-1.602	-6.001	2.797
control 40' - Protocol B' 15'	3.657	-0.7417	8.056
control 40' - Protocol B'40'	-0.2320	-4.631	4.167
control 40' - Protocol B' 70'	-0.5930	-4.992	3.806
control 40' - Protocol B' 120	-1.658	-6.057	2.741
control 70' - control 120'	-0.9500	-5.349	3.449
control 70' - 4mg/ml 15'	3.741	-0.6577	8.140
control 70' - 4mg/mL 40'	-7.843	-12.242	-3.444
control 70' - 4mg/mL 70'	-50.926	-55.325	-46.527
control 70' - 4mg/mL 120'	-57.389	-61.788	-52.990
control 70' - Protocol A 15'	2.620	-1.779	7.019
control 70' - Protocol A 40'	-0.7410	-5.140	3.658
control 70' - Protocol A 70'	-0.2500	-4.649	4.149
control 70' - Protocol A 120'	-1.833	-6.232	2.566
control 70' - Protocol B' 15'	3.426	-0.9727	7.825
control 70' - Protocol B'40'	-0.4630	-4.862	3.936
control 70' - Protocol B' 70'	-0.8240	-5.223	3.575

control 70' - Protocol B' 120	-1.889	-6.288	2.510
control 120' - 4mg/ml 15'	4.691	0.2923	9.090
control 120' - 4mg/mL 40'	-6.893	-11.292	-2.494
control 120' - 4mg/mL 70'	-49.976	-54.375	-45.577
control 120' - 4mg/mL 120'	-56.439	-60.838	-52.040
control 120' - Protocol A 15'	3.570	-0.8287	7.969
control 120' - Protocol A 40'	0.2090	-4.190	4.608
control 120' - Protocol A 70'	0.7000	-3.699	5.099
control 120' - Protocol A 120'	-0.8830	-5.282	3.516
control 120' - Protocol B' 15'	4.376	-0.02273	8.775
control 120' - Protocol B'40'	0.4870	-3.912	4.886
control 120' - Protocol B' 70'	0.1260	-4.273	4.525
control 120' - Protocol B' 120	-0.9390	-5.338	3.460
4mg/ml 15' - 4mg/mL 40'	-11.584	-15.983	-7.185
4mg/ml 15' - 4mg/mL 70'	-54.667	-59.066	-50.268
4mg/ml 15' - 4mg/mL 120'	-61.130	-65.529	-56.731
4mg/ml 15' - Protocol A 15'	-1.121	-5.520	3.278
4mg/ml 15' - Protocol A 40'	-4.482	-8.881	-0.08327
4mg/ml 15' - Protocol A 70'	-3.991	-8.390	0.4077
4mg/ml 15' - Protocol A 120'	-5.574	-9.973	-1.175
4mg/ml 15' - Protocol B' 15'	-0.3150	-4.714	4.084
4mg/ml 15' - Protocol B'40'	-4.204	-8.603	0.1947
4mg/ml 15' - Protocol B' 70'	-4.565	-8.964	-0.1663
4mg/ml 15' - Protocol B' 120	-5.630	-10.029	-1.231
4mg/mL 40' - 4mg/mL 70'	-43.083	-47.482	-38.684
4mg/mL 40' - 4mg/mL 120'	-49.546	-53.945	-45.147
4mg/mL 40' - Protocol A 15'	10.463	6.064	14.862
4mg/mL 40' - Protocol A 40'	7.102	2.703	11.501
4mg/mL 40' - Protocol A 70'	7.593	3.194	11.992
4mg/mL 40' - Protocol A 120'	6.010	1.611	10.409

4mg/mL 40' - Protocol B' 15'	11.269	6.870	15.668
4mg/mL 40' - Protocol B'40'	7.380	2.981	11.779
4mg/mL 40' - Protocol B' 70'	7.019	2.620	11.418
4mg/mL 40' - Protocol B' 120	5.954	1.555	10.353
4mg/mL 70' - 4mg/mL 120'	-6.463	-10.862	-2.064
4mg/mL 70' - Protocol A 15'	53.546	49.147	57.945
4mg/mL 70' - Protocol A 40'	50.185	45.786	54.584
4mg/mL 70' - Protocol A 70'	50.676	46.277	55.075
4mg/mL 70' - Protocol A 120'	49.093	44.694	53.492
4mg/mL 70' - Protocol B' 15'	54.352	49.953	58.751
4mg/mL 70' - Protocol B'40'	50.463	46.064	54.862
4mg/mL 70' - Protocol B' 70'	50.102	45.703	54.501
4mg/mL 70' - Protocol B' 120	49.037	44.638	53.436
4mg/mL 120' - Protocol A 15'	60.009	55.610	64.408
4mg/mL 120' - Protocol A 40'	56.648	52.249	61.047
4mg/mL 120' - Protocol A 70'	57.139	52.740	61.538
4mg/mL 120' - Protocol A 120'	55.556	51.157	59.955
4mg/mL 120' - Protocol B' 15'	60.815	56.416	65.214
4mg/mL 120' - Protocol B'40'	56.926	52.527	61.325
4mg/mL 120' - Protocol B' 70'	56.565	52.166	60.964
4mg/mL 120' - Protocol B' 120	55.500	51.101	59.899
Protocol A 15' - Protocol A 40'	-3.361	-7.760	1.038
Protocol A 15' - Protocol A 70'	-2.870	-7.269	1.529
Protocol A 15' - Protocol A 120'	-4.453	-8.852	-0.05427
Protocol A 15' - Protocol B' 15'	0.8060	-3.593	5.205
Protocol A 15' - Protocol B'40'	-3.083	-7.482	1.316
Protocol A 15' - Protocol B' 70'	-3.444	-7.843	0.9547
Protocol A 15' - Protocol B' 120	-4.509	-8.908	-0.1103
Protocol A 40' - Protocol A 70'	0.4910	-3.908	4.890
Protocol A 40' - Protocol A 120'	-1.092	-5.491	3.307

Protocol A 40' - Protocol B' 15'	4.167	-0.2317	8.566
Protocol A 40' - Protocol B'40'	0.2780	-4.121	4.677
Protocol A 40' - Protocol B' 70'	-0.08300	-4.482	4.316
Protocol A 40' - Protocol B' 120	-1.148	-5.547	3.251
Protocol A 70' - Protocol A 120'	-1.583	-5.982	2.816
Protocol A 70' - Protocol B' 15'	3.676	-0.7227	8.075
Protocol A 70' - Protocol B'40'	-0.2130	-4.612	4.186
Protocol A 70' - Protocol B' 70'	-0.5740	-4.973	3.825
Protocol A 70' - Protocol B' 120	-1.639	-6.038	2.760
Protocol A 120' - Protocol B' 15'	5.259	0.8603	9.658
Protocol A 120' - Protocol B'40'	1.370	-3.029	5.769
Protocol A 120' - Protocol B' 70'	1.009	-3.390	5.408
Protocol A 120' - Protocol B' 120	-0.05600	-4.455	4.343
Protocol B' 15' - Protocol B'40'	-3.889	-8.288	0.5097
Protocol B' 15' - Protocol B' 70'	-4.250	-8.649	0.1487
Protocol B' 15' - Protocol B' 120	-5.315	-9.714	-0.9163
Protocol B'40' - Protocol B' 70'	-0.3610	-4.760	4.038
Protocol B'40' - Protocol B' 120	-1.426	-5.825	2.973
Protocol B' 70' - Protocol B' 120	-1.065	-5.464	3.334

Intermediate calculations. ANOVA table

Source of variation	Degrees of freedom	Sum of squares	Mean square
Treatments (between columns)	15	15646	1043.1
Residuals (within columns)	32	67.407	2.106
Total	47	15714	

F = 495.18 =(MStreatment/MSresidual)

Appendix 3

Figure 3 Statistic

Table for statistic figure 3: The soluble papain (4 g x L^{-1}) or soluble papain Soluble Papain (4 g x L-1) + SDS (0.1 g x L-1) were responsible for yeast deflocculation in all recycling when compared with control (p<0.05).

	**Soluble Papain (4 g x L^{-1}) (cycle)**						**Soluble Papain (4 g x L^{-1}) + SDS (0.1 g x L^{-1}) (cycle)**					
Controle (cycle)	1st	4st	7st	10st	13st	15st	1st	4st	7st	10st	13st	15st
1st	s						s					
4st		s						s				
7st			s						s			
10st				s						s		
13st					s						s	
15st						s						s

ns – no signifcative (p>0.05), s signifcative (p<0.05).

Comparison	Mean Difference	q		P value
Control 1cicle vs Control 4cicl	-32.010	11.701	***	P<0.001
Control 1cycle vs Control 7cicl	0.4000	0.1462	ns	P>0.05
Control 1cycle vs Control 10cicl	4.440	1.623	ns	P>0.05
Control 1cycle vs Control 13cycle	-3.140	1.148	ns	P>0.05
Control 1cycle vs Control 15cic	-12.030	4.397	ns	P>0.05
Control 1cycle vs 4mg/mL 1cycle	-77.970	28.501	***	P<0.001
Control 1cycle vs 4mg/mL 4 cycle	-77.970	28.501	***	P<0.001
Control 1cycle vs 4mg/mL 7 cycle	-66.300	24.235	***	P<0.001
Control 1cycle vs 4mg/mL 10 cycle	-72.130	26.367	***	P<0.001
Control 1cycle vs 4mg/mL13cycle	-77.970	28.501	***	P<0.001
Control 1cycle vs 4mg/mL 15cycle	-40.530	14.815	***	P<0.001
Control 1cycle vs 4mg+10SDS 1ci	-77.970	28.501	***	P<0.001
Control 1cycle vs 4mg+10SDS 4cic	-77.970	28.501	***	P<0.001
Control 1cycle vs 4mg+10SDS 7cicl	-74.850	27.361	***	P<0.001
Control 1cycle vs 4mg+10SDS 10cic	-77.970	28.501	***	P<0.001
Control 1cycle vs 4mg+10SDS 13cic	-72.520	26.509	***	P<0.001
Control 1cycle vs 4mg+10SDS 15cic	-57.260	20.931	***	P<0.001

Control 4cicl vs Control 7cicl 32.410 11.847 *** P<0.001
Control 4cicl vs Control 10cicl 36.450 13.324 *** P<0.001
Control 4cicl vs Control 13cycle 28.870 10.553 *** P<0.001
Control 4cicl vs Control 15cic 19.980 7.304 *** P<0.001
Control 4cicl vs 4mg/mL 1cycle -45.960 16.800 *** P<0.001
Control 4cicl vs 4mg/mL 4 cycle -45.960 16.800 *** P<0.001
Control 4cicl vs 4mg/mL 7 cycle -34.290 12.534 *** P<0.001
Control 4cicl vs 4mg/mL 10 cycle -40.120 14.666 *** P<0.001
Control 4cicl vs 4mg/mL13cycle -45.960 16.800 *** P<0.001
Control 4cicl vs 4mg/mL 15cycle -8.520 3.114 ns P>0.05
Control 4cicl vs 4mg+10SDS 1ci -45.960 16.800 *** P<0.001
Control 4cicl vs 4mg+10SDS 4cic -45.960 16.800 *** P<0.001
Control 4cicl vs 4mg+10SDS 7cicl -42.840 15.660 *** P<0.001
Control 4cicl vs 4mg+10SDS 10cic -45.960 16.800 *** P<0.001
Control 4cicl vs 4mg+10SDS 13cic -40.510 14.808 *** P<0.001
Control 4cicl vs 4mg+10SDS 15cic -25.250 9.230 *** P<0.001
Control 7cicl vs Control 10cicl 4.040 1.477 ns P>0.05
Control 7cicl vs Control 13cycle -3.540 1.294 ns P>0.05
Control 7cicl vs Control 15cic -12.430 4.544 ns P>0.05
Control 7cicl vs 4mg/mL 1cycle -78.370 28.648 *** P<0.001
Control 7cicl vs 4mg/mL 4 cycle -78.370 28.648 *** P<0.001
Control 7cicl vs 4mg/mL 7 cycle -66.700 24.382 *** P<0.001
Control 7cicl vs 4mg/mL 10 cycle -72.530 26.513 *** P<0.001
Control 7cicl vs 4mg/mL13cycle -78.370 28.648 *** P<0.001
Control 7cicl vs 4mg/mL 15cycle -40.930 14.962 *** P<0.001
Control 7cicl vs 4mg+10SDS 1ci -78.370 28.648 *** P<0.001
Control 7cicl vs 4mg+10SDS 4cic -78.370 28.648 *** P<0.001
Control 7cicl vs 4mg+10SDS 7cicl -75.250 27.507 *** P<0.001
Control 7cicl vs 4mg+10SDS 10cic -78.370 28.648 *** P<0.001
Control 7cicl vs 4mg+10SDS 13cic -72.920 26.655 *** P<0.001
Control 7cicl vs 4mg+10SDS 15cic -57.660 21.077 *** P<0.001
Control 10cicl vs Control 13cycle -7.580 2.771 ns P>0.05
Control 10cicl vs Control 15cic -16.470 6.020 ** P<0.01
Control 10cicl vs 4mg/mL 1cycle -82.410 30.124 *** P<0.001
Control 10cicl vs 4mg/mL 4 cycle -82.410 30.124 *** P<0.001
Control 10cicl vs 4mg/mL 7 cycle -70.740 25.858 *** P<0.001
Control 10cicl vs 4mg/mL 10 cycle -76.570 27.990 *** P<0.001
Control 10cicl vs 4mg/mL13cycle -82.410 30.124 *** P<0.001
Control 10cicl vs 4mg/mL 15cycle -44.970 16.438 *** P<0.001
Control 10cicl vs 4mg+10SDS 1ci -82.410 30.124 *** P<0.001

Control 10cicl vs 4mg+10SDS 4cic	-82.410	30.124	***	P<0.001
Control 10cicl vs 4mg+10SDS 7cicl	-79.290	28.984	***	P<0.001
Control 10cicl vs 4mg+10SDS 10cic	-82.410	30.124	***	P<0.001
Control 10cicl vs 4mg+10SDS 13cic	-76.960	28.132	***	P<0.001
Control 10cicl vs 4mg+10SDS 15cic	-61.700	22.554	***	P<0.001
Control 13cycle vs Control 15cic	-8.890	3.250	ns	P>0.05
Control 13cycle vs 4mg/mL 1cycle	-74.830	27.353	***	P<0.001
Control 13cycle vs 4mg/mL 4 cycle	-74.830	27.353	***	P<0.001
Control 13cycle vs 4mg/mL 7 cycle	-63.160	23.088	***	P<0.001
Control 13cycle vs 4mg/mL 10 cycle	-68.990	25.219	***	P<0.001
Control 13cycle vs 4mg/mL13cycle	-74.830	27.353	***	P<0.001
Control 13cycle vs 4mg/mL 15cycle	-37.390	13.668	***	P<0.001
Control 13cycle vs 4mg+10SDS 1ci	-74.830	27.353	***	P<0.001
Control 13cycle vs 4mg+10SDS 4cic	-74.830	27.353	***	P<0.001
Control 13cycle vs 4mg+10SDS 7cicl	-71.710	26.213	***	P<0.001
Control 13cycle vs 4mg+10SDS 10cic	-74.830	27.353	***	P<0.001
Control 13cycle vs 4mg+10SDS 13cic	-69.380	25.361	***	P<0.001
Control 13cycle vs 4mg+10SDS 15cic	-54.120	19.783	***	P<0.001
Control 15cic vs 4mg/mL 1cycle	-65.940	24.104	***	P<0.001
Control 15cic vs 4mg/mL 4 cycle	-65.940	24.104	***	P<0.001
Control 15cic vs 4mg/mL 7 cycle	-54.270	19.838	***	P<0.001
Control 15cic vs 4mg/mL 10 cycle	-60.100	21.969	***	P<0.001
Control 15cic vs 4mg/mL13cycle	-65.940	24.104	***	P<0.001
Control 15cic vs 4mg/mL 15cycle	-28.500	10.418	***	P<0.001
Control 15cic vs 4mg+10SDS 1ci	-65.940	24.104	***	P<0.001
Control 15cic vs 4mg+10SDS 4cic	-65.940	24.104	***	P<0.001
Control 15cic vs 4mg+10SDS 7cicl	-62.820	22.963	***	P<0.001
Control 15cic vs 4mg+10SDS 10cic	-65.940	24.104	***	P<0.001
Control 15cic vs 4mg+10SDS 13cic	-60.490	22.112	***	P<0.001
Control 15cic vs 4mg+10SDS 15cic	-45.230	16.533	***	P<0.001
4mg/mL 1cycle vs 4mg/mL 4 cycle	0.000	0.000	ns	P>0.05
4mg/mL 1cycle vs 4mg/mL 7 cycle	11.670	4.266	ns	P>0.05
4mg/mL 1cycle vs 4mg/mL 10 cycle	5.840	2.135	ns	P>0.05
4mg/mL 1cycle vs 4mg/mL13cycle	0.000	0.000	ns	P>0.05
4mg/mL 1cycle vs 4mg/mL 15cycle	37.440	13.686	***	P<0.001
4mg/mL 1cycle vs 4mg+10SDS 1ci	0.000	0.000	ns	P>0.05
4mg/mL 1cycle vs 4mg+10SDS 4cic	0.000	0.000	ns	P>0.05
4mg/mL 1cycle vs 4mg+10SDS 7cicl	3.120	1.140	ns	P>0.05
4mg/mL 1cycle vs 4mg+10SDS 10cic	0.000	0.000	ns	P>0.05
4mg/mL 1cycle vs 4mg+10SDS 13cic	5.450	1.992	ns	P>0.05

4mg/mL 1cycle vs 4mg+10SDS 15cic	20.710	7.570	***	P<0.001
4mg/mL 4 cycle vs 4mg/mL 7 cycle	11.670	4.266	ns	P>0.05
4mg/mL 4 cycle vs 4mg/mL 10 cycle	5.840	2.135	ns	P>0.05
4mg/mL 4 cycle vs 4mg/mL13cycle	0.000	0.000	ns	P>0.05
4mg/mL 4 cycle vs 4mg/mL 15cycle	37.440	13.686	***	P<0.001
4mg/mL 4 cycle vs 4mg+10SDS 1ci	0.000	0.000	ns	P>0.05
4mg/mL 4 cycle vs 4mg+10SDS 4cic	0.000	0.000	ns	P>0.05
4mg/mL 4 cycle vs 4mg+10SDS 7cicl	3.120	1.140	ns	P>0.05
4mg/mL 4 cycle vs 4mg+10SDS 10cic	0.000	0.000	ns	P>0.05
4mg/mL 4 cycle vs 4mg+10SDS 13cic	5.450	1.992	ns	P>0.05
4mg/mL 4 cycle vs 4mg+10SDS 15cic	20.710	7.570	***	P<0.001
4mg/mL 7 cycle vs 4mg/mL 10 cycle	-5.830	2.131	ns	P>0.05
4mg/mL 7 cycle vs 4mg/mL13cycle	-11.670	4.266	ns	P>0.05
4mg/mL 7 cycle vs 4mg/mL 15cycle	25.770	9.420	***	P<0.001
4mg/mL 7 cycle vs 4mg+10SDS 1ci	-11.670	4.266	ns	P>0.05
4mg/mL 7 cycle vs 4mg+10SDS 4cic	-11.670	4.266	ns	P>0.05
4mg/mL 7 cycle vs 4mg+10SDS 7cicl	-8.550	3.125	ns	P>0.05
4mg/mL 7 cycle vs 4mg+10SDS 10cic	-11.670	4.266	ns	P>0.05
4mg/mL 7 cycle vs 4mg+10SDS 13cic	-6.220	2.274	ns	P>0.05
4mg/mL 7 cycle vs 4mg+10SDS 15cic	9.040	3.304	ns	P>0.05
4mg/mL 10 cycle vs 4mg/mL13cycle	-5.840	2.135	ns	P>0.05
4mg/mL 10 cycle vs 4mg/mL 15cycle	31.600	11.551	***	P<0.001
4mg/mL 10 cycle vs 4mg+10SDS 1ci	-5.840	2.135	ns	P>0.05
4mg/mL 10 cycle vs 4mg+10SDS 4cic	-5.840	2.135	ns	P>0.05
4mg/mL 10 cycle vs 4mg+10SDS 7cicl	-2.720	0.9943	ns	P>0.05
4mg/mL 10 cycle vs 4mg+10SDS 10cic	-5.840	2.135	ns	P>0.05
4mg/mL 10 cycle vs 4mg+10SDS 13cic	-0.3900	0.1426	ns	P>0.05
4mg/mL 10 cycle vs 4mg+10SDS 15cic	14.870	5.436	*	P<0.05
4mg/mL13cycle vs 4mg/mL 15cycle	37.440	13.686	***	P<0.001
4mg/mL13cycle vs 4mg+10SDS 1ci	0.000	0.000	ns	P>0.05
4mg/mL13cycle vs 4mg+10SDS 4cic	0.000	0.000	ns	P>0.05
4mg/mL13cycle vs 4mg+10SDS 7cicl	3.120	1.140	ns	P>0.05
4mg/mL13cycle vs 4mg+10SDS 10cic	0.000	0.000	ns	P>0.05
4mg/mL13cycle vs 4mg+10SDS 13cic	5.450	1.992	ns	P>0.05
4mg/mL13cycle vs 4mg+10SDS 15cic	20.710	7.570	***	P<0.001
4mg/mL 15cycle vs 4mg+10SDS 1ci	-37.440	13.686	***	P<0.001
4mg/mL 15cycle vs 4mg+10SDS 4cic	-37.440	13.686	***	P<0.001
4mg/mL 15cycle vs 4mg+10SDS 7cicl	-34.320	12.545	***	P<0.001
4mg/mL 15cycle vs 4mg+10SDS 10cic	-37.440	13.686	***	P<0.001
4mg/mL 15cycle vs 4mg+10SDS 13cic	-31.990	11.694	***	P<0.001

4mg/mL 15cycle vs 4mg+10SDS 15cic	-16.730	6.116	**	P<0.01
4mg+10SDS 1ci vs 4mg+10SDS 4cic	0.000	0.000	ns	P>0.05
4mg+10SDS 1ci vs 4mg+10SDS 7cicl	3.120	1.140	ns	P>0.05
4mg+10SDS 1ci vs 4mg+10SDS 10cic	0.000	0.000	ns	P>0.05
4mg+10SDS 1ci vs 4mg+10SDS 13cic	5.450	1.992	ns	P>0.05
4mg+10SDS 1ci vs 4mg+10SDS 15cic	20.710	7.570	***	P<0.001
4mg+10SDS 4cic vs 4mg+10SDS 7cicl	3.120	1.140	ns	P>0.05
4mg+10SDS 4cic vs 4mg+10SDS 10cic	0.000	0.000	ns	P>0.05
4mg+10SDS 4cic vs 4mg+10SDS 13cic	5.450	1.992	ns	P>0.05
4mg+10SDS 4cic vs 4mg+10SDS 15cic	20.710	7.570	***	P<0.001
4mg+10SDS 7cicl vs 4mg+10SDS 10cic	-3.120	1.140	ns	P>0.05
4mg+10SDS 7cicl vs 4mg+10SDS 13cic	2.330	0.8517	ns	P>0.05
4mg+10SDS 7cicl vs 4mg+10SDS 15cic	17.590	6.430	**	P<0.01
4mg+10SDS 10cic vs 4mg+10SDS 13cic	5.450	1.992	ns	P>0.05
4mg+10SDS 10cic vs 4mg+10SDS 15cic	20.710	7.570	***	P<0.001
4mg+10SDS 13cic vs 4mg+10SDS 15cic	15.260	5.578	*	P<0.05

	Mean	95% Confidence Interval	
Difference	Difference	From	To
================================	=========	=======	=======
Control 1cycle - Control 4cicl	-32.010	-46.049	-17.971
Control 1cycle - Control 7cicl	0.4000	-13.639	14.439
Control 1cycle - Control 10cicl	4.440	-9.599	18.479
Control 1cycle - Control 13cycle	-3.140	-17.179	10.899
Control 1cycle - Control 15cic	-12.030	-26.069	2.009
Control 1cycle - 4mg/mL 1cycle	-77.970	-92.009	-63.931
Control 1cycle - 4mg/mL 4 cycle	-77.970	-92.009	-63.931
Control 1cycle - 4mg/mL 7 cycle	-66.300	-80.339	-52.261
Control 1cycle - 4mg/mL 10 cycle	-72.130	-86.169	-58.091
Control 1cycle - 4mg/mL13cycle	-77.970	-92.009	-63.931
Control 1cycle - 4mg/mL 15cycle	-40.530	-54.569	-26.491
Control 1cycle - 4mg+10SDS 1ci	-77.970	-92.009	-63.931
Control 1cycle - 4mg+10SDS 4cic	-77.970	-92.009	-63.931
Control 1cycle - 4mg+10SDS 7cicl	-74.850	-88.889	-60.811
Control 1cycle - 4mg+10SDS 10cic	-77.970	-92.009	-63.931
Control 1cycle - 4mg+10SDS 13cic	-72.520	-86.559	-58.481
Control 1cycle - 4mg+10SDS 15cic	-57.260	-71.299	-43.221
Control 4cicl - Control 7cicl	32.410	18.371	46.449
Control 4cicl - Control 10cicl	36.450	22.411	50.489
Control 4cicl - Control 13cycle	28.870	14.831	42.909

Control 4cicl - Control 15cic	19.980	5.941	34.019
Control 4cicl - 4mg/mL 1cycle	-45.960	-59.999	-31.921
Control 4cicl - 4mg/mL 4 cycle	-45.960	-59.999	-31.921
Control 4cicl - 4mg/mL 7 cycle	-34.290	-48.329	-20.251
Control 4cicl - 4mg/mL 10 cycle	-40.120	-54.159	-26.081
Control 4cicl - 4mg/mL13cycle	-45.960	-59.999	-31.921
Control 4cicl - 4mg/mL 15cycle	-8.520	-22.559	5.519
Control 4cicl - 4mg+10SDS 1ci	-45.960	-59.999	-31.921
Control 4cicl - 4mg+10SDS 4cic	-45.960	-59.999	-31.921
Control 4cicl - 4mg+10SDS 7cicl	-42.840	-56.879	-28.801
Control 4cicl - 4mg+10SDS 10cic	-45.960	-59.999	-31.921
Control 4cicl - 4mg+10SDS 13cic	-40.510	-54.549	-26.471
Control 4cicl - 4mg+10SDS 15cic	-25.250	-39.289	-11.211
Control 7cicl - Control 10cicl	4.040	-9.999	18.079
Control 7cicl - Control 13cycle	-3.540	-17.579	10.499
Control 7cicl - Control 15cic	-12.430	-26.469	1.609
Control 7cicl - 4mg/mL 1cycle	-78.370	-92.409	-64.331
Control 7cicl - 4mg/mL 4 cycle	-78.370	-92.409	-64.331
Control 7cicl - 4mg/mL 7 cycle	-66.700	-80.739	-52.661
Control 7cicl - 4mg/mL 10 cycle	-72.530	-86.569	-58.491
Control 7cicl - 4mg/mL13cycle	-78.370	-92.409	-64.331
Control 7cicl - 4mg/mL 15cycle	-40.930	-54.969	-26.891
Control 7cicl - 4mg+10SDS 1ci	-78.370	-92.409	-64.331
Control 7cicl - 4mg+10SDS 4cic	-78.370	-92.409	-64.331
Control 7cicl - 4mg+10SDS 7cicl	-75.250	-89.289	-61.211
Control 7cicl - 4mg+10SDS 10cic	-78.370	-92.409	-64.331
Control 7cicl - 4mg+10SDS 13cic	-72.920	-86.959	-58.881
Control 7cicl - 4mg+10SDS 15cic	-57.660	-71.699	-43.621
Control 10cicl - Control 13cycle	-7.580	-21.619	6.459
Control 10cicl - Control 15cic	-16.470	-30.509	-2.431
Control 10cicl - 4mg/mL 1cycle	-82.410	-96.449	-68.371
Control 10cicl - 4mg/mL 4 cycle	-82.410	-96.449	-68.371
Control 10cicl - 4mg/mL 7 cycle	-70.740	-84.779	-56.701
Control 10cicl - 4mg/mL 10 cycle	-76.570	-90.609	-62.531
Control 10cicl - 4mg/mL13cycle	-82.410	-96.449	-68.371
Control 10cicl - 4mg/mL 15cycle	-44.970	-59.009	-30.931
Control 10cicl - 4mg+10SDS 1ci	-82.410	-96.449	-68.371
Control 10cicl - 4mg+10SDS 4cic	-82.410	-96.449	-68.371
Control 10cicl - 4mg+10SDS 7cicl	-79.290	-93.329	-65.251
Control 10cicl - 4mg+10SDS 10cic	-82.410	-96.449	-68.371

Control 10cicl - 4mg+10SDS 13cic	-76.960	-90.999	-62.921
Control 10cicl - 4mg+10SDS 15cic	-61.700	-75.739	-47.661
Control 13cycle - Control 15cic	-8.890	-22.929	5.149
Control 13cycle - 4mg/mL 1cycle	-74.830	-88.869	-60.791
Control 13cycle - 4mg/mL 4 cycle	-74.830	-88.869	-60.791
Control 13cycle - 4mg/mL 7 cycle	-63.160	-77.199	-49.121
Control 13cycle - 4mg/mL 10 cycle	-68.990	-83.029	-54.951
Control 13cycle - 4mg/mL13cycle	-74.830	-88.869	-60.791
Control 13cycle - 4mg/mL 15cycle	-37.390	-51.429	-23.351
Control 13cycle - 4mg+10SDS 1ci	-74.830	-88.869	-60.791
Control 13cycle - 4mg+10SDS 4cic	-74.830	-88.869	-60.791
Control 13cycle - 4mg+10SDS 7cicl	-71.710	-85.749	-57.671
Control 13cycle - 4mg+10SDS 10cic	-74.830	-88.869	-60.791
Control 13cycle - 4mg+10SDS 13cic	-69.380	-83.419	-55.341
Control 13cycle - 4mg+10SDS 15cic	-54.120	-68.159	-40.081
Control 15cic - 4mg/mL 1cycle	-65.940	-79.979	-51.901
Control 15cic - 4mg/mL 4 cycle	-65.940	-79.979	-51.901
Control 15cic - 4mg/mL 7 cycle	-54.270	-68.309	-40.231
Control 15cic - 4mg/mL 10 cycle	-60.100	-74.139	-46.061
Control 15cic - 4mg/mL13cycle	-65.940	-79.979	-51.901
Control 15cic - 4mg/mL 15cycle	-28.500	-42.539	-14.461
Control 15cic - 4mg+10SDS 1ci	-65.940	-79.979	-51.901
Control 15cic - 4mg+10SDS 4cic	-65.940	-79.979	-51.901
Control 15cic - 4mg+10SDS 7cicl	-62.820	-76.859	-48.781
Control 15cic - 4mg+10SDS 10cic	-65.940	-79.979	-51.901
Control 15cic - 4mg+10SDS 13cic	-60.490	-74.529	-46.451
Control 15cic - 4mg+10SDS 15cic	-45.230	-59.269	-31.191
4mg/mL 1cycle - 4mg/mL 4 cycle	0.000	-14.039	14.039
4mg/mL 1cycle - 4mg/mL 7 cycle	11.670	-2.369	25.709
4mg/mL 1cycle - 4mg/mL 10 cycle	5.840	-8.199	19.879
4mg/mL 1cycle - 4mg/mL13cycle	0.000	-14.039	14.039
4mg/mL 1cycle - 4mg/mL 15cycle	37.440	23.401	51.479
4mg/mL 1cycle - 4mg+10SDS 1ci	0.000	-14.039	14.039
4mg/mL 1cycle - 4mg+10SDS 4cic	0.000	-14.039	14.039
4mg/mL 1cycle - 4mg+10SDS 7cicl	3.120	-10.919	17.159
4mg/mL 1cycle - 4mg+10SDS 10cic	0.000	-14.039	14.039
4mg/mL 1cycle - 4mg+10SDS 13cic	5.450	-8.589	19.489
4mg/mL 1cycle - 4mg+10SDS 15cic	20.710	6.671	34.749
4mg/mL 4 cycle - 4mg/mL 7 cycle	11.670	-2.369	25.709
4mg/mL 4 cycle - 4mg/mL 10 cycle	5.840	-8.199	19.879

4mg/mL 4 cycle - 4mg/mL13cycle	0.000	-14.039	14.039
4mg/mL 4 cycle - 4mg/mL 15cycle	37.440	23.401	51.479
4mg/mL 4 cycle - 4mg+10SDS 1ci	0.000	-14.039	14.039
4mg/mL 4 cycle - 4mg+10SDS 4cic	0.000	-14.039	14.039
4mg/mL 4 cycle - 4mg+10SDS 7cicl	3.120	-10.919	17.159
4mg/mL 4 cycle - 4mg+10SDS 10cic	0.000	-14.039	14.039
4mg/mL 4 cycle - 4mg+10SDS 13cic	5.450	-8.589	19.489
4mg/mL 4 cycle - 4mg+10SDS 15cic	20.710	6.671	34.749
4mg/mL 7 cycle - 4mg/mL 10 cycle	-5.830	-19.869	8.209
4mg/mL 7 cycle - 4mg/mL13cycle	-11.670	-25.709	2.369
4mg/mL 7 cycle - 4mg/mL 15cycle	25.770	11.731	39.809
4mg/mL 7 cycle - 4mg+10SDS 1ci	-11.670	-25.709	2.369
4mg/mL 7 cycle - 4mg+10SDS 4cic	-11.670	-25.709	2.369
4mg/mL 7 cycle - 4mg+10SDS 7cicl	-8.550	-22.589	5.489
4mg/mL 7 cycle - 4mg+10SDS 10cic	-11.670	-25.709	2.369
4mg/mL 7 cycle - 4mg+10SDS 13cic	-6.220	-20.259	7.819
4mg/mL 7 cycle - 4mg+10SDS 15cic	9.040	-4.999	23.079
4mg/mL 10 cycle - 4mg/mL13cycle	-5.840	-19.879	8.199
4mg/mL 10 cycle - 4mg/mL 15cycle	31.600	17.561	45.639
4mg/mL 10 cycle - 4mg+10SDS 1ci	-5.840	-19.879	8.199
4mg/mL 10 cycle - 4mg+10SDS 4cic	-5.840	-19.879	8.199
4mg/mL 10 cycle - 4mg+10SDS 7cicl	-2.720	-16.759	11.319
4mg/mL 10 cycle - 4mg+10SDS 10cic	-5.840	-19.879	8.199
4mg/mL 10 cycle - 4mg+10SDS 13cic	-0.3900	-14.429	13.649
4mg/mL 10 cycle - 4mg+10SDS 15cic	14.870	0.8306	28.909
4mg/mL13cycle - 4mg/mL 15cycle	37.440	23.401	51.479
4mg/mL13cycle - 4mg+10SDS 1ci	0.000	-14.039	14.039
4mg/mL13cycle - 4mg+10SDS 4cic	0.000	-14.039	14.039
4mg/mL13cycle - 4mg+10SDS 7cicl	3.120	-10.919	17.159
4mg/mL13cycle - 4mg+10SDS 10cic	0.000	-14.039	14.039
4mg/mL13cycle - 4mg+10SDS 13cic	5.450	-8.589	19.489
4mg/mL13cycle - 4mg+10SDS 15cic	20.710	6.671	34.749
4mg/mL 15cycle - 4mg+10SDS 1ci	-37.440	-51.479	-23.401
4mg/mL 15cycle - 4mg+10SDS 4cic	-37.440	-51.479	-23.401
4mg/mL 15cycle - 4mg+10SDS 7cicl	-34.320	-48.359	-20.281
4mg/mL 15cycle - 4mg+10SDS 10cic	-37.440	-51.479	-23.401
4mg/mL 15cycle - 4mg+10SDS 13cic	-31.990	-46.029	-17.951
4mg/mL 15cycle - 4mg+10SDS 15cic	-16.730	-30.769	-2.691
4mg+10SDS 1ci - 4mg+10SDS 4cic	0.000	-14.039	14.039
4mg+10SDS 1ci - 4mg+10SDS 7cicl	3.120	-10.919	17.159

4mg+10SDS 1ci - 4mg+10SDS 10cic	0.000	-14.039	14.039
4mg+10SDS 1ci - 4mg+10SDS 13cic	5.450	-8.589	19.489
4mg+10SDS 1ci - 4mg+10SDS 15cic	20.710	6.671	34.749
4mg+10SDS 4cic - 4mg+10SDS 7cicl	3.120	-10.919	17.159
4mg+10SDS 4cic - 4mg+10SDS 10cic	0.000	-14.039	14.039
4mg+10SDS 4cic - 4mg+10SDS 13cic	5.450	-8.589	19.489
4mg+10SDS 4cic - 4mg+10SDS 15cic	20.710	6.671	34.749
4mg+10SDS 7cicl - 4mg+10SDS 10cic	-3.120	-17.159	10.919
4mg+10SDS 7cicl - 4mg+10SDS 13cic	2.330	-11.709	16.369
4mg+10SDS 7cicl - 4mg+10SDS 15cic	17.590	3.551	31.629
4mg+10SDS 10cic - 4mg+10SDS 13cic	5.450	-8.589	19.489
4mg+10SDS 10cic - 4mg+10SDS 15cic	20.710	6.671	34.749
4mg+10SDS 13cic - 4mg+10SDS 15cic	15.260	1.221	29.299

Intermediate calculations. ANOVA table

Source of variation	Degrees of freedom	Sum of squares	Mean square
Treatments (between columns)	17	93397	5493.9
Residuals (within columns)	72	2694.2	37.419
Total	89	96091	

F = 146.82 =(MStreatment/MSresidual)

Appendix 4

Table 4:

Comparison	Difference	q		P value
Control vs Sulfuric Acid	4.810	10.816	***	P<0.001
Control vs Papain	-0.4000	0.8995	ns	P>0.05
Sulfuric Acid vs Papain	-5.210	11.716	***	P<0.001

Intermediate calculations. ANOVA table

Source of variation	Degrees of freedom	Sum of squares	Mean square
Treatments (between columns)	2	84.067	42.034
Residuals (within columns)	12	11.866	0.9888
Total	14	95.933	

F = 42.510 =(MStreatment/MSresidual)

Appendix 5

Table 5: glutaraldehyde

Comparison	Difference	q		P value
control vs 0.1	1.860	119.63	***	P<0.001
control vs 0.5	1.910	122.84	***	P<0.001
control vs 1.0	1.920	123.49	***	P<0.001
control vs 1.5	1.920	123.49	***	P<0.001
control vs 2.0	1.930	124.13	***	P<0.001
control vs 5.0	2.030	130.56	***	P<0.001
control vs 10	2.040	131.20	***	P<0.001
0.1 vs 0.5	0.05000	3.216	ns	P>0.05
0.1 vs 1.0	0.06000	3.859	ns	P>0.05
0.1 vs 1.5	0.06000	3.859	ns	P>0.05
0.1 vs 2.0	0.07000	4.502	ns	P>0.05
0.1 vs 5.0	0.1700	10.934	***	P<0.001
0.1 vs 10	0.1800	11.577	***	P<0.001
0.5 vs 1.0	0.01000	0.6432	ns	P>0.05
0.5 vs 1.5	0.01000	0.6432	ns	P>0.05
0.5 vs 2.0	0.02000	1.286	ns	P>0.05
0.5 vs 5.0	0.1200	7.718	**	P<0.01
0.5 vs 10	0.1300	8.361	***	P<0.001
1.0 vs 1.5	0.000	0.000	ns	P>0.05
1.0 vs 2.0	0.010000	0.6432	ns	P>0.05
1.0 vs 5.0	0.1100	7.075	**	P<0.01
1.0 vs 10	0.1200	7.718	**	P<0.01
1.5 vs 2.0	0.010000	0.6432	ns	P>0.05
1.5 vs 5.0	0.1100	7.075	**	P<0.01
1.5 vs 10	0.1200	7.718	**	P<0.01
2.0 vs 5.0	0.1000	6.432	**	P<0.01
2.0 vs 10	0.1100	7.075	**	P<0.01
5.0 vs 10	0.01000	0.6432	ns	P>0.05

	Mean	95% Confidence Interval	
Difference	Difference	From	To
control - 0.1	1.860	1.784	1.936
control - 0.5	1.910	1.834	1.986
control - 1.0	1.920	1.844	1.996
control - 1.5	1.920	1.844	1.996
control - 2.0	1.930	1.854	2.006
control - 5.0	2.030	1.954	2.106
control - 10	2.040	1.964	2.116
0.1 - 0.5	0.05000	-0.02614	0.1261
0.1 - 1.0	0.06000	-0.01614	0.1361
0.1 - 1.5	0.06000	-0.01614	0.1361

0.1 - 2.0	0.07000	-0.006140	0.1461
0.1 - 5.0	0.1700	0.09386	0.2461
0.1 - 10	0.1800	0.1039	0.2561
0.5 - 1.0	0.01000	-0.06614	0.08614
0.5 - 1.5	0.01000	-0.06614	0.08614
0.5 - 2.0	0.02000	-0.05614	0.09614
0.5 - 5.0	0.1200	0.04386	0.1961
0.5 - 10	0.1300	0.05386	0.2061
1.0 - 1.5	0.000	-0.07614	0.07614
1.0 - 2.0	0.010000	-0.06614	0.08614
1.0 - 5.0	0.1100	0.03386	0.1861
1.0 - 10	0.1200	0.04386	0.1961
1.5 - 2.0	0.010000	-0.06614	0.08614
1.5 - 5.0	0.1100	0.03386	0.1861
1.5 - 10	0.1200	0.04386	0.1961
2.0 - 5.0	0.1000	0.02386	0.1761
2.0 - 10	0.1100	0.03386	0.1861
5.0 - 10	0.01000	-0.06614	0.08614

Intermediate calculations. ANOVA table

Source of variation	Degrees of freedom	Sum of squares	Mean square
Treatments (between columns)	7	10.002	1.429
Residuals (within columns)	16	0.01160	0.0007253
Total	23	10.013	

F = 1970.1 =(MStreatment/MSresidual)

Table 5: Polyethyleneimine

Comparison	Mean Difference	q	P value
control vs 0.1	-0.3300	3.868	ns P>0.05
control vs 0.5	0.4300	5.040	* P<0.05
control vs 1.0	1.720	20.160	*** P<0.001
control vs 1.5	1.870	21.918	*** P<0.001
control vs 2.0	2.100	24.614	*** P<0.001
control vs 5.0	1.940	22.738	*** P<0.001
control vs 10	2.090	24.497	*** P<0.001
0.1 vs 0.5	0.7600	8.908	*** P<0.001
0.1 vs 1.0	2.050	24.028	*** P<0.001
0.1 vs 1.5	2.200	25.786	*** P<0.001
0.1 vs 2.0	2.430	28.482	*** P<0.001
0.1 vs 5.0	2.270	26.606	*** P<0.001

0.1 vs 10	2.420	28.364	***	P<0.001
0.5 vs 1.0	1.290	15.120	***	P<0.001
0.5 vs 1.5	1.440	16.878	***	P<0.001
0.5 vs 2.0	1.670	19.574	***	P<0.001
0.5 vs 5.0	1.510	17.698	***	P<0.001
0.5 vs 10	1.660	19.457	***	P<0.001
1.0 vs 1.5	0.1500	1.758	ns	P>0.05
1.0 vs 2.0	0.3800	4.454	ns	P>0.05
1.0 vs 5.0	0.2200	2.579	ns	P>0.05
1.0 vs 10	0.3700	4.337	ns	P>0.05
1.5 vs 2.0	0.2300	2.696	ns	P>0.05
1.5 vs 5.0	0.07000	0.8205	ns	P>0.05
1.5 vs 10	0.2200	2.579	ns	P>0.05
2.0 vs 5.0	-0.1600	1.875	ns	P>0.05
2.0 vs 10	-0.01000	0.1172	ns	P>0.05
5.0 vs 10	0.1500	1.758	ns	P>0.05

	Mean	95% Confidence Interval	
Difference	Difference	From	To
control - 0.1	-0.3300	-0.7478	0.08780
control - 0.5	0.4300	0.01220	0.8478
control - 1.0	1.720	1.302	2.138
control - 1.5	1.870	1.452	2.288
control - 2.0	2.100	1.682	2.518
control - 5.0	1.940	1.522	2.358
control - 10	2.090	1.672	2.508
0.1 - 0.5	0.7600	0.3422	1.178
0.1 - 1.0	2.050	1.632	2.468
0.1 - 1.5	2.200	1.782	2.618
0.1 - 2.0	2.430	2.012	2.848
0.1 - 5.0	2.270	1.852	2.688
0.1 - 10	2.420	2.002	2.838
0.5 - 1.0	1.290	0.8722	1.708
0.5 - 1.5	1.440	1.022	1.858
0.5 - 2.0	1.670	1.252	2.088
0.5 - 5.0	1.510	1.092	1.928
0.5 - 10	1.660	1.242	2.078
1.0 - 1.5	0.1500	-0.2678	0.5678
1.0 - 2.0	0.3800	-0.03780	0.7978
1.0 - 5.0	0.2200	-0.1978	0.6378
1.0 - 10	0.3700	-0.04780	0.7878
1.5 - 2.0	0.2300	-0.1878	0.6478
1.5 - 5.0	0.07000	-0.3478	0.4878
1.5 - 10	0.2200	-0.1978	0.6378
2.0 - 5.0	-0.1600	-0.5778	0.2578
2.0 - 10	-0.01000	-0.4278	0.4078
5.0 - 10	0.1500	-0.2678	0.5678

Intermediate calculations. ANOVA table

Source of	Degrees of	Sum of	Mean

variation	freedom	squares	square
Treatments (between columns)	7	21.710	3.101
Residuals (within columns)	16	0.3494	0.02184
Total	23	22.060	

F = 142.02 =(MStreatment/MSresidual)

Table 5 : TPP

Comparison	Mean Difference	q	P value
control vs 0.1	0.9300	20.214	*** P<0.001
control vs 0.5	1.140	24.779	*** P<0.001
control vs 1.0	1.180	25.648	*** P<0.001
control vs 1.5	1.730	37.603	*** P<0.001
control vs 2.0	2.060	44.776	*** P<0.001
control vs 5.0	2.130	46.297	*** P<0.001
control vs 10	2.270	49.340	*** P<0.001
0.1 vs 0.5	0.2100	4.564	ns P>0.05
0.1 vs 1.0	0.2500	5.434	* P<0.05
0.1 vs 1.5	0.8000	17.389	*** P<0.001
0.1 vs 2.0	1.130	24.561	*** P<0.001
0.1 vs 5.0	1.200	26.083	*** P<0.001
0.1 vs 10	1.340	29.126	*** P<0.001
0.5 vs 1.0	0.04000	0.8694	ns P>0.05
0.5 vs 1.5	0.5900	12.824	*** P<0.001
0.5 vs 2.0	0.9200	19.997	*** P<0.001
0.5 vs 5.0	0.9900	21.518	*** P<0.001
0.5 vs 10	1.130	24.561	*** P<0.001
1.0 vs 1.5	0.5500	11.955	*** P<0.001
1.0 vs 2.0	0.8800	19.127	*** P<0.001
1.0 vs 5.0	0.9500	20.649	*** P<0.001
1.0 vs 10	1.090	23.692	*** P<0.001
1.5 vs 2.0	0.3300	7.173	** P<0.01
1.5 vs 5.0	0.4000	8.694	*** P<0.001
1.5 vs 10	0.5400	11.737	*** P<0.001
2.0 vs 5.0	0.07000	1.521	ns P>0.05
2.0 vs 10	0.2100	4.564	ns P>0.05
5.0 vs 10	0.1400	3.043	ns P>0.05

Difference	Mean Difference	95% Confidence Interval From	To
control - 0.1	0.9300	0.7047	1.155
control - 0.5	1.140	0.9147	1.365
control - 1.0	1.180	0.9547	1.405
control - 1.5	1.730	1.505	1.955
control - 2.0	2.060	1.835	2.285
control - 5.0	2.130	1.905	2.355

control - 10	2.270	2.045	2.495
0.1 - 0.5	0.2100	-0.01530	0.4353
0.1 - 1.0	0.2500	0.02470	0.4753
0.1 - 1.5	0.8000	0.5747	1.025
0.1 - 2.0	1.130	0.9047	1.355
0.1 - 5.0	1.200	0.9747	1.425
0.1 - 10	1.340	1.115	1.565
0.5 - 1.0	0.04000	-0.1853	0.2653
0.5 - 1.5	0.5900	0.3647	0.8153
0.5 - 2.0	0.9200	0.6947	1.145
0.5 - 5.0	0.9900	0.7647	1.215
0.5 - 10	1.130	0.9047	1.355
1.0 - 1.5	0.5500	0.3247	0.7753
1.0 - 2.0	0.8800	0.6547	1.105
1.0 - 5.0	0.9500	0.7247	1.175
1.0 - 10	1.090	0.8647	1.315
1.5 - 2.0	0.3300	0.1047	0.5553
1.5 - 5.0	0.4000	0.1747	0.6253
1.5 - 10	0.5400	0.3147	0.7653
2.0 - 5.0	0.07000	-0.1553	0.2953
2.0 - 10	0.2100	-0.01530	0.4353
5.0 - 10	0.1400	-0.08530	0.3653

Intermediate calculations. ANOVA table

Source of variation	Degrees of freedom	Sum of squares	Mean square
Treatments (between columns)	7	12.372	1.767
Residuals (within columns)	16	0.1016	0.006350
Total	23	12.474	

F = 278.34 =(MStreatment/MSresidual)
